# Structure-Activity Relationship Studies of *β*-Lactam-azide Analogues as Orally Active Antitumor Agents Targeting the Tubulin Colchicine Site

**DOI:** 10.1038/s41598-017-12912-4

**Published:** 2017-10-06

**Authors:** Dong-Jun Fu, Ling Fu, Ying-Chao Liu, Jun-Wei Wang, Yu-Qing Wang, Bing-Kai Han, Xiao-Rui Li, Chuang Zhang, Feng Li, Jian Song, Bing Zhao, Ruo-Wang Mao, Ruo-Han Zhao, Sai-Yang Zhang, Li Zhang, Yan-Bing Zhang, Hong-Min Liu

**Affiliations:** School of Pharmaceutical Sciences & Collaborative Innovation Center of New Drug Research and Safety Evaluation, Zhengzhou University, Zhengzhou, 450001 China

## Abstract

We have synthesized a series of new *β*-lactam-azide derivatives as orally active anti-tumor agents by targeting tubulin colchicine binding site and examined their structure activity relationship (SAR). Among them, compound **28** exhibited the most potent antiproliferative activity against MGC-803 cells with an IC_50_ value of 0.106 μM by induction of G2/M arrest and apoptosis and inhibition of the epithelial to mesenchymal transition. **28** acted as a novel inhibitor of tubulin polymerization by its binding to the colchicine site. SAR analysis revealed that a hydrogen atom at the C-3 position of the *β*-lactam was required for the potent antiproliferative activity of *β*-lactam-azide derivatives. Oral administration of compound **28** also effectively inhibited MGC-803 xenograft tumor growth *in vivo* in nude mice without causing significant loss of body weight. These results suggested that compound **28** is a promising orally active anticancer agent with potential for development of further clinical applications.

## Introduction

Microtubules, filamentous cytoskeleton protein polymers composed of *α-* and *β-*tubulin heterodimers, are vital components of all cells and play diverse roles in a variety of essential cellular processes including maintenance of cell structure, protein trafficking, chromosomal segregation, and mitosis^1–3^. Microtubule targeting agents are known to interact with tubulin through at least four binding sites: the laulimalide site, paclitaxel site, vinblastine site, and colchicine site^4^. As one of the tubulin-targeting agents, colchicine binding site inhibitors exert their biological effects by inhibiting tubulin assembly and suppressing microtubule formation^5–9^. A water-soluble phosphate prodrug CA-4P targeting colchicine binding site has FDA-designated orphan drug status for the treatment of anaplastic thyroid cancer and ovarian cancer^10^. However, neural and cardiovascular toxicities of CA-4P currently represent the main obstacle to broad its clinical application in different cancers^11^. Therefore, there is still a need to develop new inhibitors of tubulin polymerization by targeting the colchicine binding site for cancer therapy.

*β*-Lactam skeleton has attracted much attention from medicinal chemists for many years because of their numerous biological activities^12,13^, especially their antitumor activity^14–16^. Apart from their pharmacological use, *β*-lactams have been used as synthons in the preparation of various heterocyclic compounds of biological significance^17^. For example, suitably substituted hydroxyl *β*-lactam has been used in the semisynthesis of a side chain bridged paclitaxel^18^. Importantly, some *β*-lactam analogues were shown to cause apoptosis in cancer cells through induction of microtubule disorganization and mitotic catastrophe^19,20^. Hence, in this study, *β*-lactam was chosen as a basic skeleton to develop new antitumor agents.

**Figure 1:**
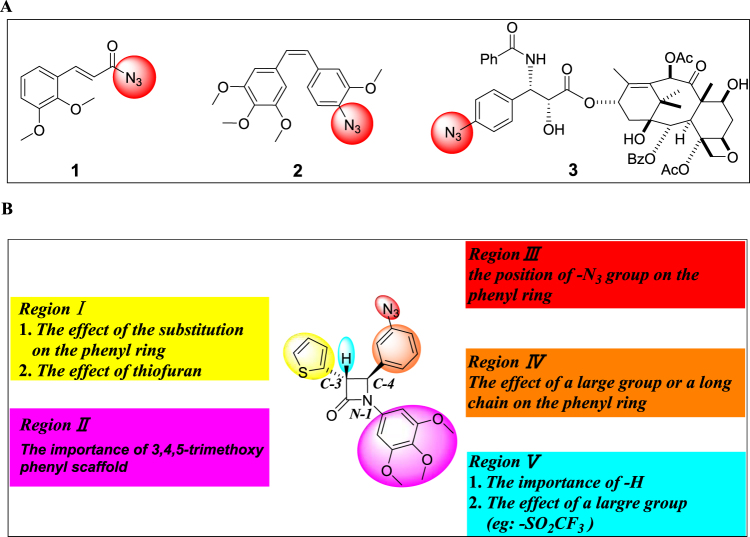
(**A**) Azide derivatives as anticancer agents. (**B**) Five key regions (I-V) to explore detailed structure activity relationships of *β*-lactam-azides.

In addition, azide moiety has been widely used as a scaffold to design new chemical entities for anti-proliferation^21–26^ and arylazide derivatives have been shown to be potent antitumor agents. For example, *trans*-2,3-dimethoxycinnamoyl azide derivative **1** (Fig. 1A) enhanced the *in vitro* and *in vivo* antitumor effect of romidepsin on bladder cancer cells^27^. The Combretastatin A-4 aryl azide analogue **2** (Fig. 1A) displayed a potent anti-tubulin activity with an IC_50_ value of 5.2 μM^28^. 3′-(4-Azidophenyl)-3′-dephenylpaclitaxel **3** (Fig. 1A) was developed as a novel paclitaxel photoaffinity probe and shown to be as active as paclitaxel in tubulin assembly and cytotoxicity assays^29^.

These intriguing findings and our continuous quest to identify more potent antitumor candidates^30–32^ led us to design novel *β*-lactam and azide hybrids. In this work, a series of *β*-lactam-azide derivatives as tubulin polymerization inhibitors were synthesized and evaluated their antitumor activity *in vitro* and *in vivo*. In addition, the detailed structure activity relationships in five regions of *β*-lactam-azides were explored (Fig. 1B) to provide further insight for developing more efficient tubulin targeting and antiproliferative agents for cancer therapy.

## Results and Discussion

### Chemistry

**Figure 2:**
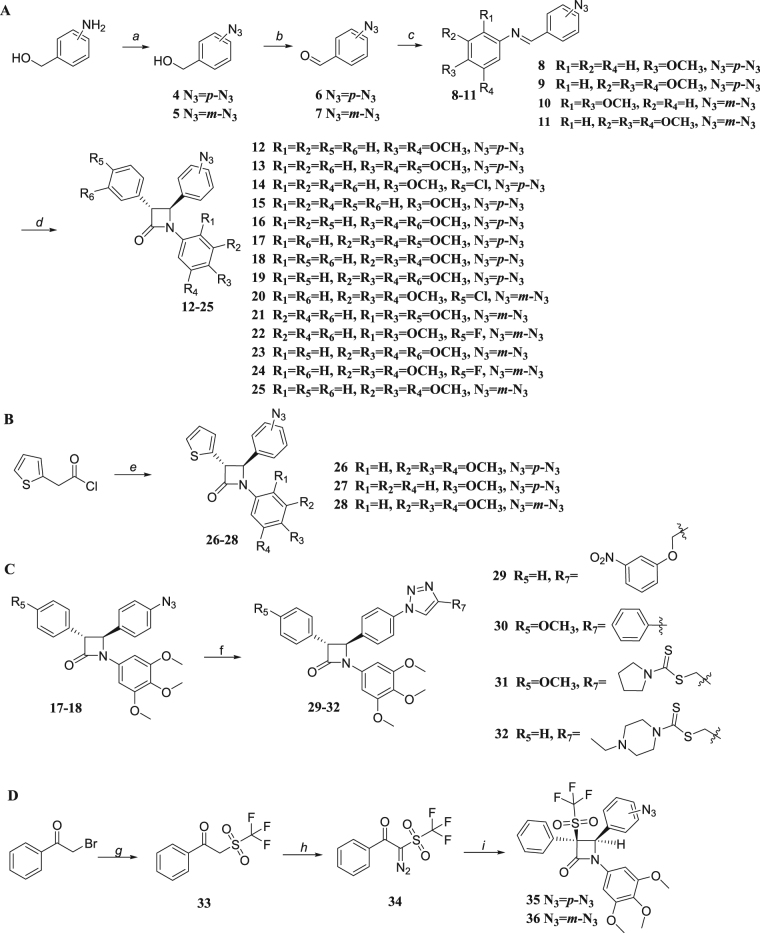
Reagents and conditions: (**a**) NaNO_2_, 2 M H_2_SO_4_, 0 °C, 30 min, NaN_3_, rt, 2 h; (**b**) PCC, CH_2_Cl_2_, rt, 2 h; (**c**) EtOH, reflux; (**d**) substituted phenylacetyl chloride, triethylamine, anhydrous CH_2_Cl_2_, reflux, 3~8 h; (**e**) triethylamine, imines **8–9** or **11**, anhydrous CH_2_Cl_2_, MgSO_4_, reflux, 3~12 h; (**f**) CuSO_4_.5H_2_O, VcNa, THF-H_2_O, rt, 12 h; (**g**) CF_3_NaO_2_S, DMAC, N_2_, 50 °C, 14 h; (**h**) TfN_3_, CH_3_CN, pyridine, 0 °C, 16 h; (**i**) Substituted imine, toluene, N_2_, 100 °C, 2 h.

The synthetic routes of the desired novel *β*-lactam-azide derivatives were outlined in Fig. 2. Synthesis of *β*-lactams **12–28** was carried out using Staudinger reaction with *in situ* generation of a ketene and subsequent reaction with the appropriately substituted imines^33^. The *trans* stereochemistry was observed for azetidin-2-one derivatives **12–28** with aromatic rings directly attached to positions 3 and 4 of the *β*-lactam scaffold, as evidenced by the coupling constants, *J*_*3,4*_ ≈ 2.4 Hz. No *cis* isomers (*J*_*3,4*_ ≈ 5 Hz) were detected in this series, possibly due to steric hindrance between the 3- and 4-positions of the *β*-lactam ring (Fig. 2A,B). To explore the effect of a large group or a long chain on the phenyl ring at the C-4 position of the *β*-lactam, 1,2,3-triazole analogues **29–32** in Fig. 2C as ring-closing products were synthesized through a Huisgen 1,3-dipolar cycloaddition^34^. *β*-Lactams-triflones **35–36** were prepared via a Staudinger [2 + 2] cycloaddition of imines with aryl trifly ketene generated *in situ* from 2-diazo-1-phenyl-2-(trifluoromethylsulfonyl) ethanone **34** by a Wolff rearrangement in satisfactory to good yields from the reported procedure^35^. An X-ray crystallography study of the *β*-lactam products was undertaken to confirm the stereochemical assignments and explore possible important structural features for potent activity. ORTEP diagram for compound **28** (CCDC number: 1526687) was presented (see Supplementary Fig. S1).

### Biology

#### Structure Activity Relationships

All synthesized *β*-lactam-azide derivatives were evaluated for their antiproliferative activity against three cancer cell lines (MGC-803, MCF-7, A549) using CCK-8 proliferation assay. The well-known anticancer drug CA-4P was used as a control^36,37^. The results were summarized in Table 1.

**Table 1:** IC_50_ values (μM) of synthesized compounds.

Compd.	IC_50_ (μM)		
	MGC-803	MCF-7	A549
12	>20	>20	>20
13	>20	>20	>20
14	>20	>20	>20
15	>20	>20	>20
16	>20	>20	>20
17	0.590 ± 0.014	1.075 ± 0.024	3.218 ± 0.349
18	0.171 ± 0.051	0.338 ± 0.004	1.739 ± 0.145
19	1.395 ± 0.133	2.882 ± 0.489	18.130 ± 2.310
20	3.113 ± 0.373	4.959 ± 0.427	4.382 ± 0.483
21	>20	>20	>20
22	>20	>20	>20
23	1.401 ± 0.127	2.880 ± 0.494	18.134 ± 1.712
24	0.840 ± 0.630	3.997 ± 0.922	5.763 ± 0.887
25	0.392 ± 0.028	0.805 ± 0.176	2.130 ± 0.390
26	0.251 ± 0.053	0.154 ± 0.019	4.203 ± 0.186
27	>20	>20	>20
28	0.106 ± 0.001	0.421 ± 0.047	0.507 ± 0.052
29	>20	>20	>20
30	>20	>20	>20
31	>20	>20	>20
32	>20	>20	>20
35	>20	>20	>20
36	>20	>20	>20
CA-4P	0.015 ± 0.001	0.023 ± 0.002	0.033 ± 0.007

Cell lines were treated with compounds for 48 h. IC_50_ values were indicated as the mean ± SD (standard error) of at least three independent experiments.

In a series of analogues **12**–**25**, we mainly investigated the effects of substitution on the phenyl ring at the C-3 position of the *β*-lactam (Region I), the importance of 3,4,5-trimethoxyphenyl group as the *β*-lactam N-1 substituent (Region II). During the SAR studies, we found that the substitution on the phenyl ring at the C-3 position of the *β*-lactam was important for the activity showing over 8-fold activity loss against the growth of MGC-803 cells, when the hydrogen atom (**18**) was replaced with the methoxy group (**19**). *β*-Lactam-azide derivatives **12–16** and **21–22** without 3,4,5-trimethoxyphenyl group at the N-1 position of the *β*-lactam displayed relatively lower antiproliferative activity (IC_50_ > 20 μM) toward three cancer cell lines, indicating that the 3,4,5-trimethoxyphenyl group at the N-1 position was crucial for their antiproliferative activity.

To investigate whether the heterocycle displayed an effect on the antiproliferative activity (Region I), compounds with a thiofuran ring (**26–28**) at the C-3 position of the *β*-lactam were synthesized. Replacing the phenyl scaffold at the C-3 position of the *β*-lactam with a thiofuran ring led to an increased activity (compound **25** *vs*. **28**), indicating the importance of thiofuran at the C-3 position of *β*-lactam for their antiproliferative activity. We also explored the relationship between the location of azide group and their antiproliferative activity (Region III). Compound **26**, which contained -N_3_ at the *para*-position, gave the IC_50_ values of 0.154~4.203 μM toward three cancer cell lines. Interestingly, when the -N_3_ was moved to the *meta*-position, the obtained compound **28** displayed the better activity (IC_50_ values of 0.106–0.507 μM). Thus, the location of -N_3_ group on the phenyl ring at the C-4 position of the *β*-lactam displayed a significantly improved antiproliferative activity to the cancer cell lines.

Furthermore, *β*-lactam-1,2,3-triazoles **29**–**32** were synthesized to evaluate the importance of azide group and the effect of a large group or a long chain on the phenyl ring at the C-4 position of the *β*-lactam (Region IV). When an azide group was replaced by a large group (1,2,3-triazole) or a long chain (1,2,3-triazole-dithiocarbamate), the inhibitory activity of analogues **29**–**32** was completely lost. Those results suggested that a large group or a long chain on the phenyl ring at the C-4 position of the *β*-lactam was unfavorable for antiproliferative activity.

Because trifluoromethanesulfonyl moiety was an active group in some anticancer agents, it was often used as a promising scaffold for drug discovery^38–41^. To complete the SAR study, the effects of hydrogen atom and a large group (eg: −SO_2_CF_3_) as the *β*-lactam C-3 substituent were investigated (Region V). Changing the hydrogen atom at the C-3 position of the *β*-lactam to the trifluoromethanesulfonyl group resulted in inactive compounds **35–36**, indicating that the hydrogen atom was critical for antiproliferative activity. The detailed structure activity relationships of all the synthesized *β*-lactam-azide derivatives were summarized as Supplementary Fig. S2.

#### Compound 28 induces G2/M arrest in cell cycle progression

**Figure 3:**
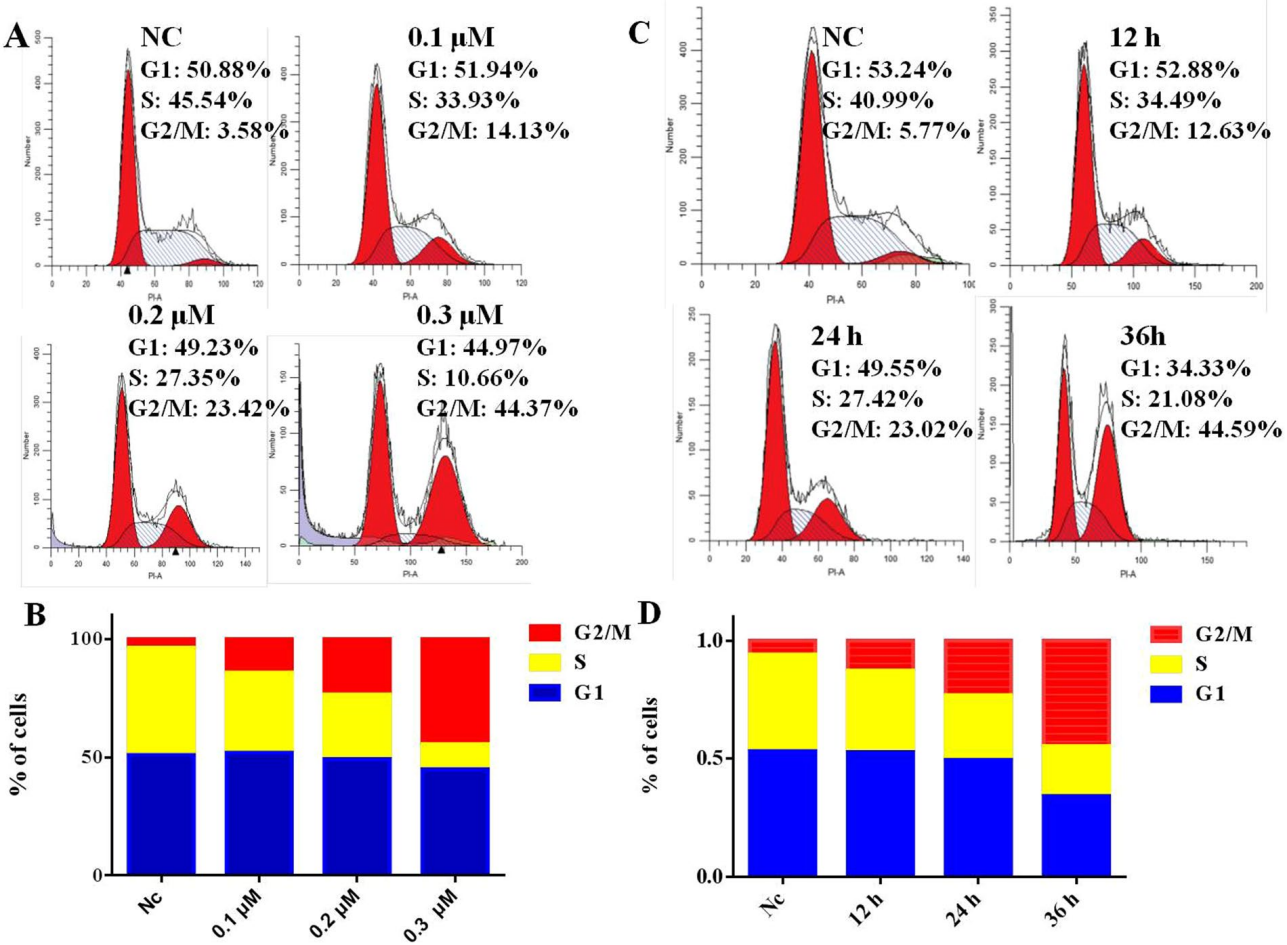
(**A**,**B**) MGC-803 Cells were treated with **28** at 0 μM, 0.1 μM, 0.2 μM and 0.3 μM for 24 h. (**C**,**D**) MGC-803 cells were treated with **28** at the indicated concentration (0.1 μM) for 0, 12, 24, and 36 h.

Due to the most potent antiproliferative activity against all selected tumor cells, compound **28** was chosen to further investigate its underlying biological mechanisms^42^. As shown in Fig. 3, **28** induced cell cycle arrest at the G2/M phase in a concentration and time dependent manner. **28** treatment of MGC-803 cells at concentrations of 0, 0.1, 0.2 and 0.3 μM for 24 h resulted in 3.58%, 14.13%, 23.42% and 44.37% of G2/M populations, respectively (Fig. 3A). when MGC-803 cells were exposed to 0.1 μM **28** for 0, 12, 24, and 36 h, the percentages of MGC-803 cells at the G2/M phase were 5.77%, 12.63%, 23.02% and 44.59%, respectively (Fig. 3C).

#### Compound 28 induces cell apoptosis by increasing the expression of BAX and decreasisng the expression of Bcl-2 leading to activation of the caspase cascade

**Figure 4:**
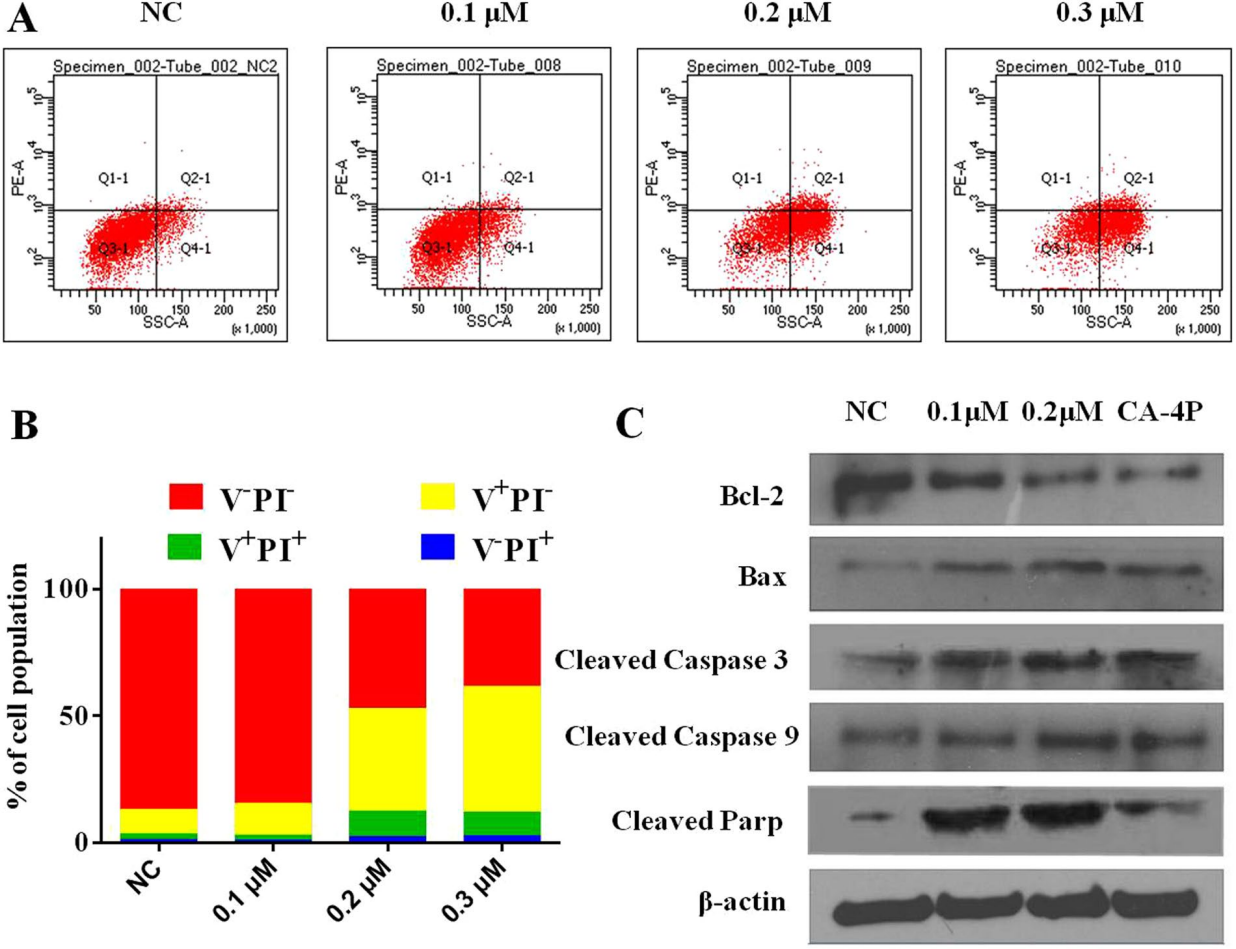
(**A**,**B**) The apoptotic effects of **28** on MGC-803 cells at 0.1, 0.2 and 0.3 μM concentrations. (**C**) Western blotting analysis of apoptosis-related proteins in **28** and CA-4P (0.01 μM) treated MGC-803 cells.

We next evaluated whether **28** induces apoptosis in MGC-803 cells by flow cytometry analysis of propidium iodide (PI) and Annexin V stained cells^43^. As shown in Fig. 4, **28** caused cell apoptosis in a concentration-dependent manner. When MGC-803 cells were incubated with **28** at 0.1, 0.2, and 0.3 μM for 24 h, the percentages of early apoptotic cells were 12.4%, 40.5%, and 49.7%, respectively and those of late apoptotic cells 2.1%, 10.1%, and 9.4%, respectively (Fig. 4A). Bcl-2 family proteins were crucial components of mitochondrial stress-induced cellular apoptosis^44^. Thus, the expression of apoptosis-related proteins was also determined. Western blotting analysis of **28** treated MGC-803 cells further revealed an increased protein expression of BAX and a decreased expression of Bcl-2, which was accompanied by increased expression of cleaved caspase-3, caspase-9 and PARP in a concentration-dependent manner (Fig. 4C).

#### Compound 28 induces tubulin destabilization targeting the colchicine site

**Figure 5:**
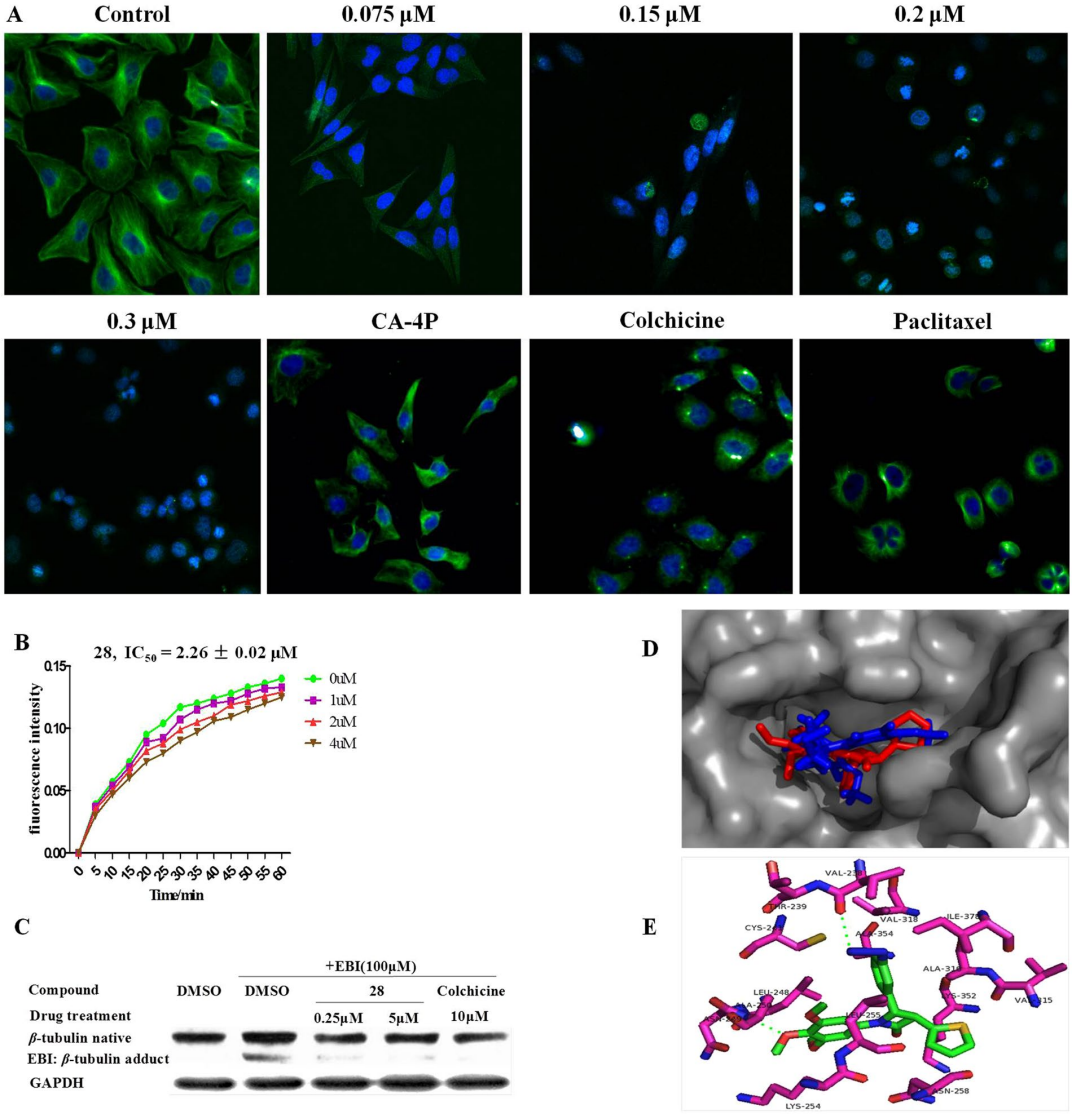
(**A**) Immunofluorescence Staining of Tubulin. MGC-803 cells were plated in culture dishes and incubated with **28** at the indicated concentrations (0, 0.075, 0.15, 0.2 and 0.3 μM), 0.004 μM CA-4P, 0.030 μM Colchicine and 0.004 μM Paclitaxel for 24 h. (**B**) Inhibition of Tubulin Polymerization assay. (**C**) EBI competition assay on MGC-803 cells. (**D**) Molecular modeling study, superimposition of the compound **28** (Red) and DAMA-colchicine (Blue) within the colchicine-binding site (PDB code 1SA0). (**E**) ligand-protein interactions of **28**.

As the microtubule system plays a vital role in the maintenance of cell shape and basic cellular functions, an immunofluorescence staining assay was performed to study whether compound **28** could disrupt the microtubule dynamics in living cells^45^. 0.075 μM **28** moderately depolymerized interphase microtubules, whereas the depolymerization effect of 0.3 μM **28** is much stronger in MGC-803 cells (Fig. 5A).

The *in vitro* tubulin polymerization inhibitory activity of **28** was then evaluated. Purified and unpolymerized tubulin was incubated with **28** at indicated concentrations, and tubulin polymerization was measured by the method originally described by D. Bone *et al*.^46^. Derivative **28** inhibited *in vitro* polymerization of a concentration dependent manner (Fig. 5B), with an IC_50_ value of 2.262 ± 0.017 μM.

In order to evaluate whether **28** directly binds to tubulin at the colchicine binding site, we carried out a competition assay with *N*,*N*′-ethylene-bis(iodoacetamide) (EBI) in MGC-803 cells as described in a previously published paper^47^. EBI was an alkylating agent that cross-links the Cys239 and the Cys354 residues of *β*-tubulin involved in the colchicine-binding site, forming a EBI: *β*-tubulin adduct^48^. The adduct was easily detectable by Western blot as a second immunoreactive *β*-tubulin band that migrated faster than *β*-tubulin itself^49^. Preincubation of **28** (0.25 and 5 μM) dose-dependently prevented the formation of the EBI: *β*-tubulin adduct, resulting in the disappearance of the adduct band, which was consistent with the effect of colchicine (10 μM). Thus, the assay (Fig. 5C) indicated that **28** may directly bind to the cochicine-binding site of *β*-tubulin.

In continuation with our efforts to rationalize our experimental findings and investigate the potential binding site of the target compound with tubulin-microtubule system, molecular modeling studies were performed as described previously^50^. Docking studies in Fig. 5D and E showed that **28** occupied the colchicine binding site of tubulin in agreement with the X-ray structure of tubulin cocrystallized with a colchicines derivative, *N*-deacetyl-*N*-(2-mercaptoacetyl)colchicine (DAMA-colchicine, PDB entry 1SA0)^51^. Compound **28** formed hydrophobic interactions with the residues of Val318, Val315, Leu248, Leu255, Ile378, Ala316, Ala354. The azide group of phenyl ring at the C-4 position of **28** formed a hydrogen bond with the residue Val238. Importantly, the *p*-methoxy group of phenyl ring at the N-1 position of the *β*-lactam **28** formed a hydrogen bond with the residue Ala250, which could explain the importance of trimethoxyphenyl ring for its potent antiproliferative activity.

#### Compound 28 inhibits migration of MGC-803 cells by up-regulation of E-cadherin and ZO-1 expression and down-regulation of N-cadherin expression

**Figure 6:**
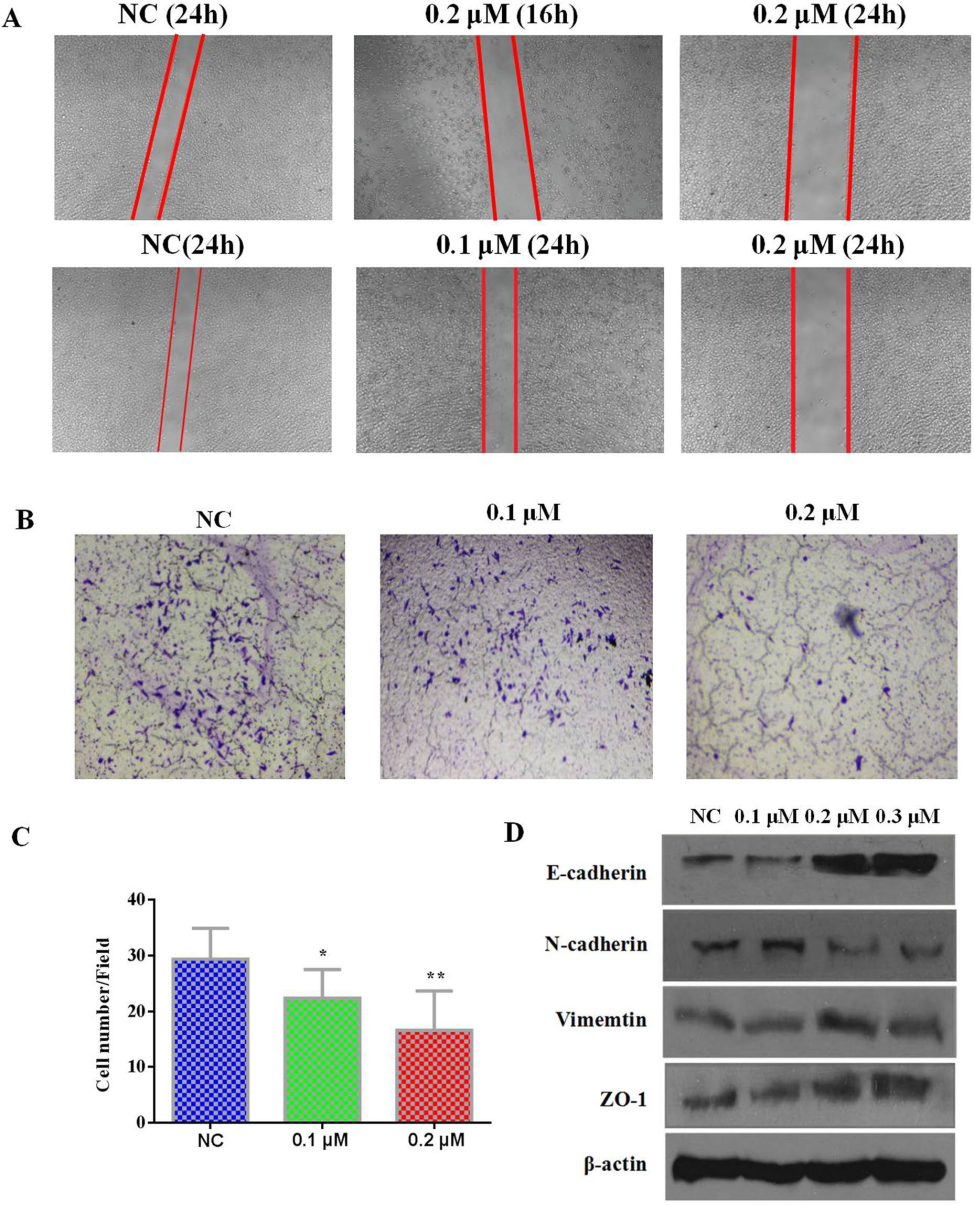
(**A**) Scarification test of **28** on MGC-803 cells. (**B**,**C**) Transwell test of **28** on MGC-803 cells. The data were presented as the mean ± SEM *P < 0.05, **P < 0.01. (**D**) MGC-803 cells were harvested and lysed for the detection of EMT-related markers after treated by different concentrations of **28**.

The epithelial- mesenchymal transition (EMT) was an unique process for the phenotypic changes of tumor cells characterized by a transition from polarized rigid epithelial cells to migrant mesenchymal cells, thus conferring the ability of tumor invasion and metastasis^52^. EMT could suppress tubulin tyrosine ligaseand promote microtubule stability^53^, resulting in tubulin detyrosination and the formation of microtentacles for supporting endothelial cell attachment^54^. In this study, we found that **28** could reverse the EMT progress (Fig. 6).

In a scarification test, compared to control, the distances of scratches after MGC-803 cells were treated with **28** obviously increased in a time-dependent and concentration-dependent manner (Fig. 6A). In a transwell test, the average numbers of migrated cells in fields of control, 0.1 and 0.2 μM **28** treated MGC-803 cells were 29.4, 22.4 and 16.6, respectively (Fig. 6B and C). Based on the results from these tests, we next examined the protein expression of EMT-related makers such as E-cadherenin, N-cadherenin, ZO-1 and Vementin^55^. The results showed that the expression of E-cadherin and ZO-1 was up-regulated and that N-cadherence and vementin were down-regulated by **28** (Fig. 6D). This result suggests that **28** could inhibit the EMT process in tumor cells.

#### The *in vivo* antitumor effect of 28 in a xenograft model

**Figure 7:**
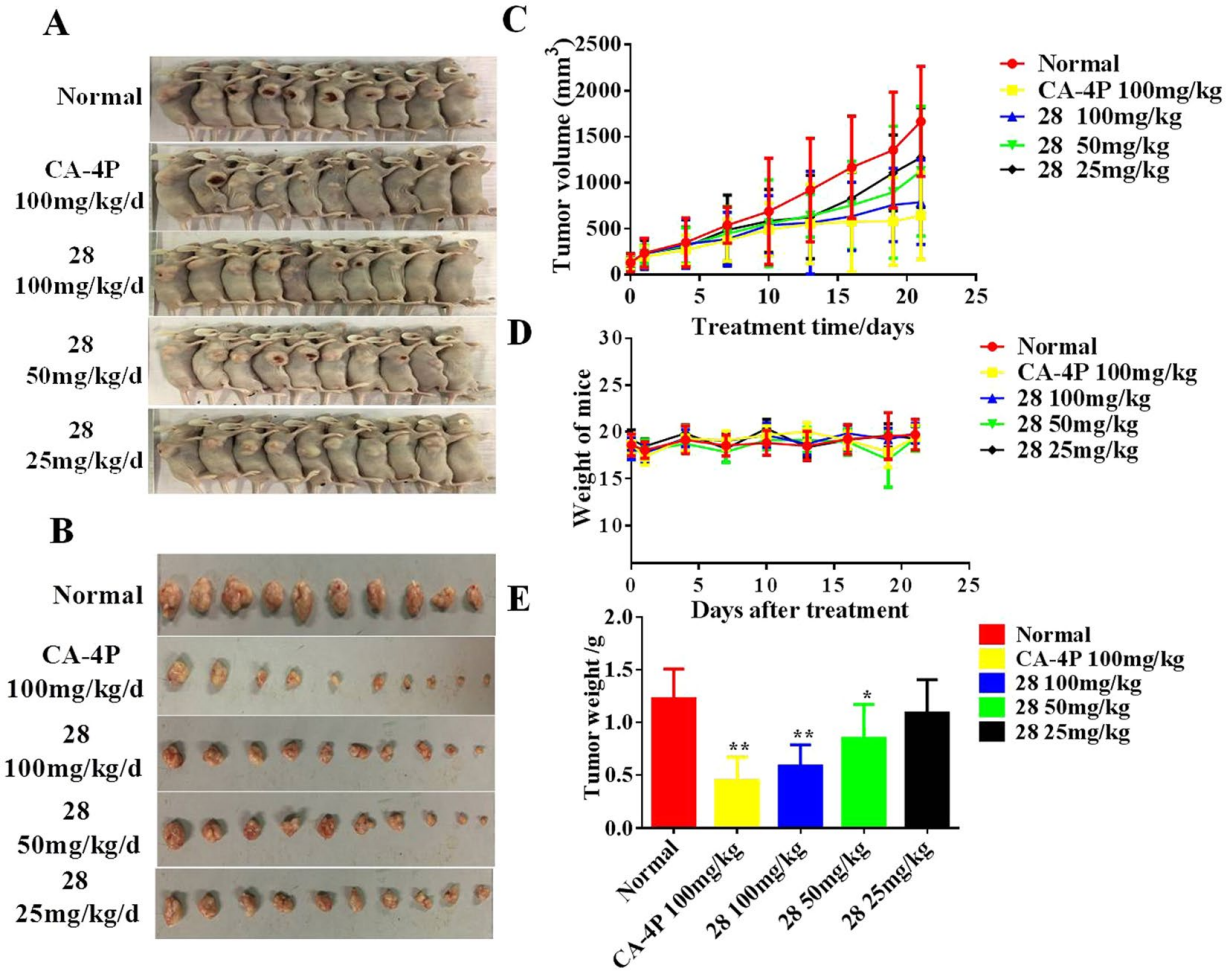
The *in vivo* antitumor activity of **28**. After administered with control (saline), CA-4P, 25, 50, and 100 mg/kg **28** for 21 days, the mice were sacrificed, and the tumors were weighed. (**A**,**B**) The images of euthanized mice and excised tumors. (**C**) Tumor volumes of mice in each group. (**D**) Body weights of mice from each group at the end of the observation period. (**E**) The weights of excised tumors from each group. The data were presented as the mean ± SEM *P < 0.05, **P < 0.01, significantly different compared with the control by test.

To evaluate the potential antitumor effects of **28** *in vivo*, a MGC-803 xenograft model were established in nude mice by subcutaneously injecting MGC-803 cells at its logarithmic phase into the right flank of mice^56^. Tumor bearing mice were then randomly assigned to five groups (control, 100 mg/kg CA-4P, 25, 50, 100 mg/kg **28**) with 10 mice per group. Then, the mice were gavaged with saline (control), CA-4P and different concentrations of **28** in saline solution daily. The results in Fig. 7 showed that 100 mg/kg **28** caused a considerable suppression of tumor growth. At the end of the observation period, mean tumor volumes of control, CA-4P, 25, 50, and 100 mg/kg **28** groups were 1665.98 ± 568.36 mm^3^, 642.61 ± 449.92 mm^3^, 788.18 ± 435.92 mm^3^, 1125.93 ± 668.25 mm^3^ and 1273.88 ± 513.69 mm^3^, respectively. The average tumor weights of control, CA-4P, 25, 50, and 100 mg/kg **28** groups were 1.23 ± 0.28 g, 0.45 ± 0.22 g (inhibitory rate: 63.27%), 0.53 ± 0.20 g (inhibitory rate: 59.34%), 0.85 ± 0.32 g (inhibitory rate: 30.78%), and 1.09 ± 0.31 g (inhibitory rate: 10.99%), respectively. The antitumor activity of **28** *in vivo* was similar to that of CA-4P. Importantly, the *in vivo* antitumor efficacy of **28** was achieved without causing any obvious loss of body weight (Fig. 7D). This result suggests that **28** has low toxicity toward mice.

## Conclusion

A series of *β*-lactam-azide derivatives were designed, synthesized and evaluated for their antitumor activities. Among them, compound **28** possessed the most potent antiproliferative ability with an IC_50_ value of 0.106 μM against MGC-803 cells.

The first SAR for this *β*-lactam-azide scaffold was explored and highlighted as follows: (1) 3,4,5-trimethoxy phenyl ring at the N-1 position of the *β*-lactam was essential; (2) a hydrogen atom at the C-3 position of the *β*-lactam was required for their potent antiproliferative activity, the large group (e.g.: –SO_2_CF_3_) at the same position diminished the inhibitory activity; and (3) the large group 1,2,3-triazole and long chain 1,2,3-triazole-dithiocarbamate on the phenyl ring at the C-4 position of the *β*-lactam completely diminished its antiproliferative activity.

Preliminary mode of action studies demonstrated that **28** halted cell cycle progression at the G2/M phase and induced apoptosis in MGC-803 cells via increased expression of BAX and decreased expression of Bcl-2. Immunofluorescence staining, *in vitro* tubulin polymerization inhibition and EBI competition assays, as well as molecular modeling study identified that compound **28** was a novel tubulin polymerization inhibitor probably by binding to the colchicine site of tubulin. For the first time, we reported that **28** inhibited cell migration by inhibiting the EMT process in gastric cancer cells. Importantly, **28** inhibited *in vivo* tumor growth in a xenograft model without apparent toxicity. The antitumor efficacy of **28** in a xenograft model of MGC-803 cells is close to that of a FDA approved anti-tubulin drug, CA-4P. Taken together, compound **28** could be a lead candidate for its further development in treatment of gastric cancer.

## Experiment Section

### Chemistry section

(The detailed information is in Supplementary Information)

### Biological section

#### Cell-based cytotoxicity screening assay

MGC-803 cell line was obtained from the Chinese Academy of Sciences (Shanghai, China). MCF-7 and A549 were from the National Cell Center, China. MGC-803 cells were cultured in DMEM culture medium (DMEM, Biological Industries, Kibbutz Beit Haemek, Israel). A549 and MCF-7 cells were cultured in 1640 medium (1640, Biological Industries, Kibbutz Beit Haemek, Israel). All medium were supplemented with 10% fetal bovine serum, 100 U/ml penicillin (North China Pharmaceutical group Co.), and 100 µg/ml streptomycin (North China Pharmaceutical group Co.). All cells were cultured at 37 °C in a humidified incubator containing 5% CO_2_.

Synthesized analogues used in the study were dissolved in 100% cell culture grade DMSO and the final concentration of DMSO as <0.1% for testing on cancer cell lines. Cytotoxicity assays were performed on the human gastric carcinoma cell line MGC-803, the human mammary carcinoma cell line MCF-7, the human lung carcinoma cell line A549. Cells (3500–5000/well) were seeded into 96-well plates in 100 µL of culture medium. The cells were treated in triplicate with a gradient concentration of testing compounds and incubated at 37 °C, 5% CO_2_ for 48 h. For all cell lines, CCK-8 assay was performed to measure cytotoxic effects. The absorbance was measured using a microplate reader (BioTek Instrument, Inc. Vermont, USA) with a test wavelength of 450 nm. The absorbance levels were corrected against untreated control absorbance values. All experiments were performed in triplicate and SPSS17.0 was used for data analysis to obtain IC_50_ values.

#### Cell cycle analysis by flow cytometry

MGC-803 cells were incubated in the absence and presence of **28**. Subsequently, the cells were fixed with 70% alcohol in PBS. The fixed cells were incubated with RNase (1 µg/mL) propidium iodide (50 µg/mL) for 2 h. Flow cytometry analysis was performed using BD FACS (Becton Dickinson, San Jose, CA, USA).

#### Analysis of apoptosis

An Annexin V-FITC/PI kit (KeyGEN BioTECH, Nanjing, China) was used to detect apoptosis. Cells were seeded in 6-well plates and treated with 0, 0.1, 0.2 and 0.3 µM of **28** for 24 h. Then MGC-803 cells were collected and suspended in binding buffer containing Annexin V-FITC (0.5 mg/mL) and PI (0.5 mg/mL) and incubated in dark for 20 min and analyzed by flow cytometry (Becton, Dickinson and Company, NJ). The apoptosis related proteins of western blot analysis were conducted according to our previously reported method^31,32^.

#### Immunostaining and microscopy

Cell climbing slices were sterilized and placed on the bottom of a 24-well plate for 24 h before MGC-803 cells were incubated in DMSO, different concentrations of **28** or CA-4P solutions were added. The next day the cell climbing slices were fixed by 4% paraformaldehyde for 15 min after washed by PBS for 3 times. 0.5% Triton-X-100 was added and shaked for 20 min. 0.1% BSA was used to block for 30 min and then removed. The cell climbing slices were added *α*-tubulin antibody (1:100) and incubated overnight at 4 °C in humid box. On the third day the humid box was taken out and balanced at 37 °C for 30 min. The cell climbing slices were washed by PBST for 3 times for each of 3 minutes and coated with FITC antibody (1:500) in a dark place for 1 h at room temperature. DAPI was used to re-stained for 5 min and then removed. The cell climbing slices were sealing by an anti-fluorescence quenching agent and images collected by Laser scanning confocal microscope (Nikon, Japan).

#### *In vitro* tubulin polymerization assay

An amount of 5.6 mg/ml tubulin was resuspended in PEM buffer [80 mM PIPES (pH 6.9), 1 mM EGTA, 0.5 mM MgCl_2_, 1 mM ATP, 10.2% (v/v) glycerol] and then was preincubated with compound **28** or vehicle DMSO on ice. The reaction was monitored by a spectrophotometer in absorbance at 340 nm at 37 °C every 5 min. The final concentrations of **28** were listed as follows: 0, 1, 2, and 4 µM.

#### EBI competition assay

Six-well plates were seeded with MGC-803 cells at 5 × 10^5^ cells per well. Cells were first incubated with compound **28** (0.25 and 5 µM), or colchicine (10 µM) for 2 h and afterward treated with EBI (100 µM). After 2 h, the cells were harvested and cell extracts were prepared for Western blot analysis. 20 μg of proteins was subjected to gel electrophoresis using 10% polyacrylamide gels. The proteins were transferred onto PVDF membranes, then blocked by 5% nonfatmilk for 1 h, and subsequently incubated with anti-*β*-tubulin antibody for 16 h at 4 °C. Next, the membranes were washed extensively and immunoreactive proteins were finally detected by chemiluminescence.

#### Molecular modeling Studies

We investigated the binding modes of the target compound by molecular docking study. For the receptor preparation, the PDB entry 1SA0 was downloaded from the Protein Data Bank (PDB). The 3D structures of the ligand **28** were generated using Chembio3D Ultra 11.0 followed by energy minimization. AutoDock 4.0 program equipped with ADT was used to perform the automated molecular docking^50^. A total of 60 possible binding conformations were generated and grouped into clusters based on a 1.0 Å cluster tolerance. The docking models were analyzed and represented using ADT.

#### Cell scarification assay

MGC-803 cells were seeded in 6-well plate until cells grew to confluence. Tips were used to make a scratch on cells. Control and **28** contained culture media without fetal bovine serum were added subsequently after 3 times of washing by PBS. Then the cells were cultured at 37 °C in a humidified incubator containing 5% CO_2_ and photos taken at 0, 16, 24 and 48 h, respectively.

#### Transwell cell migration assay

The test was performed in a transwell plate (Corning). MGC-803 cells were added to the upper chamber of a transwell plate. Control and different concentrations of **28** solutions were added to both upper and bottom chambers. Then the transwell plate was cultured at 37 °C in a humidified incubator containing 5% CO_2_ for 24 h. The next day, all contents in the upper chamber were removed and wiped by cotton buds. Alcohol was used to fix, and the crystal violet was used to stain the transwell for 30 min. After washed by PBS the cell chambers were observed and photos taken. The cell number of the migrated cells through the transwell was calculated by counting 5 visual fields of each group (P < 0.05)^55,56^.

#### *In vivo* anti-tumor activity

Animals were treated according to protocols established by the ethics committee of Zhengzhou University and the *in vivo* experiments were carried out in accordance with the approved guidelines and approved by the ethics committee of Zhengzhou University. BALB/c nude mice (18 g, aged 4–5 weeks) were purchased from Human SJA Laboratory Animal Co. Ltd. (Hunan, China). Mice were subcutaneously implanted with MGC-803 cells (5 × 10^6^ cells per mouse) on the right flank of nude mice. Once tumor volumes reached to approximately 100 mm^3^, the mice were randomly divided into corresponding saline, CA-4P (100 mg/kg), **28** (100 mg/kg), **28** (50 mg/kg) and **28** (25 mg/kg) treatment groups (n = 10 mice for each group). The treatment groups received intragastric administration of **28** and CA-4P per day for a period of 21 days. Then, the mice were euthanized and tumors isolated and weighed. Their body weights were measured and tumor sizes determined by vernier caliper measurement every other day.

#### Statistical evaluation

Data were presented as means ± SD. Statistical analyses were performed by the analysis of variance (ANOVA). All statistical analyses were performed by SPSS 17.0.

## Electronic supplementary material

Supplementary information
