# Supplementary material for: Structure-Activity Relationship Studies of β-Lactam-azide Analogues as Orally Active Antitumor Agents Targeting the Tubulin Colchicine Site
Source: Sci Rep. 2017 Oct 6;7:12788. doi: 10.1038/s41598-017-12912-4 (PMC5630639; doi:10.1038/s41598-017-12912-4)
Supplement: Supplementary file 1 — Supplementary information [file 41598_2017_12912_MOESM1_ESM.docx]

**Structure-Activity Relationship Studies of *β*-Lactam-azide Analogues as Orally Active Antitumor Agents Targeting the Tubulin Colchicine Site**

Dong-Jun Fu^1,a^, Ling Fu^1,a^, Ying-Chao Liu^1^, Jun-Wei Wang^1^, Yu-Qing Wang^1^, Bing-Kai Han^1^, Xiao-Rui Li^1^, Chuang Zhang^1^, Feng Li^1^, Jian Song^1^, Bing Zhao^1^, Ruo-Wang Mao^1^, Ruo-Han Zhao^1^, Sai-Yang Zhang^1^, Li Zhang^1^, Yan-Bing Zhang^1,*^, and Hong-Min Liu^1,*^

^1^School of Pharmaceutical Sciences & Collaborative Innovation Center of New Drug Research and Safety Evaluation, Zhengzhou University, Zhengzhou, 450001, China.

^a^These authors contributed equally to this work.

^*^Correspondence and requests for materials should be addressed to Y.-B.Z. (email: [zhangyb@zzu.edu.cn](mailto:zhangyb@zzu.edu.cn)) and H.-M.L. (email: [liuhm@zzu.edu.cn](mailto:liuhm@zzu.edu.cn)).

1. **X-Ray crystallographic structures of compound 28**
2. **Structure activity relationships of β-lactam-azide derivatives**
3. **Docking models**
4. **Figures in manuscript**
5. **General methods of chemistry section**
6. **^1^H and ^13^C NMR Spectra of all β-lactam-azide derivatives**
7. **References**
8. **X-Ray crystallographic structures of compound 28**

An X-ray crystallography study of the *β*-lactam products was undertaken to confirm the stereochemical assignments and explore possible important structural features for potent activity. ORTEP diagram for compound **28** (CCDC number: 1526687) was presented in **Fig. S1**.

**Figure S1**. Crystal structure of **28**.

1. **Structure activity relationships of *β*-lactam-azide derivatives**

Five regions of structure activity relationships were explored. The detailed illustration for structure activity relationships of all the synthesized *β*-lactam derivatives were summarized in **Fig. S2**.

**Figure S2**. Summarized SARs of synthesized compounds.

1. **Docking models**

**Figure S3**. Molecular docking was performed using a reported procedure^1^. (left) Superimposition of the compound **28** (Red) and DAMA-colchicine (Blue) within the colchicine-binding site (PDB code 1SA0)^2^. (right) ligand-protein interactions of **28**.

1. **Figures in manuscript**

**Figure 1**. (**A**) Azide derivatives as anticancer agents. (**B**) Five key regions (Ⅰ-Ⅴ) to explore detailed structure activity relationships of *β*-lactam-azides.

**Figure 2**. Reagents and conditions: (*a*) NaNO_2_, 2 M H_2_SO_4_, 0 °C, 30 min, NaN_3_, rt, 2h; (*b*) PCC, CH_2_Cl_2_, rt, 2h; (*c*) EtOH, reflux; (*d*) substituted phenylacetyl chloride, triethylamine, anhydrous CH_2_Cl_2_, reflux, 3~8 h; (*e*) triethylamine, imines **8-9** or **11**, anhydrous CH_2_Cl_2_, MgSO_4_, reflux, 3~12 h; (*f*) CuSO_4_.5H_2_O, VcNa, THF-H_2_O, rt, 12 h; (*g*) CF_3_NaO_2_S, DMAC, N_2_, 50°C, 14h; (*h*) TfN_3_, CH_3_CN, pyridine, 0 °C, 16h; (*i*) Substituted imine, toluene, N_2_, 100 °C, 2h.

**Figure 3**. (**A** and **B**) MGC-803 Cells were treated with **28** at 0 μM, 0.1 μM, 0.2 μM and 0.3 μM for 24 h. (**C** and **D**) MGC-803 cells were treated with **28** at the indicated concentration (0.1 μM) for 0, 12, 24, and 36 h.

**Figure 4.** (**A** and **B**) The apoptotic effects of **28** on MGC-803 cells at 0.1, 0.2 and 0.3 μM concentrations. (**C**) Western blotting analysis of apoptosis-related proteins in **28** and CA-4P (0.01 μM) treated MGC-803 cells.

**Figure 5**. (**A**) Immunofluorescence Staining of Tubulin. MGC-803 cells were plated in culture dishes and incubated with **28** at the indicated concentrations (0, 0.075, 0.15, 0.2 and 0.3 μM)、0.004 μM CA-4P、0.030 μM Colchicine and 0.004 μM Paclitaxel for 24 h. (**B**) Inhibition of Tubulin Polymerization assay. (**C**) EBI competition assay on MGC-803 cells. (**D**) Molecular modeling study, superimposition of the compound **28** (Red) and DAMA-colchicine (Blue) within the colchicine-binding site (PDB code 1SA0). (**E**) ligand-protein interactions of **28**.

**Figure 6**. (**A**) Scarification test of **28** on MGC-803 cells. (**B** and **C**) Transwell test of **28** on MGC-803 cells. The data were presented as the mean ± SEM *P<0.05, **P<0.01. (**D**) MGC-803 cells were harvested and lysed for the detection of EMT-related markers after treated by different concentrations of **28**.

**Figure 7.** The *in vivo* antitumor activity of **28**. After administered with control (saline), CA-4P, 25, 50, and 100 mg/kg **28** for 21 days, the mice were sacriﬁced, and the tumors were weighed. (**A** and **B**) The images of euthanized mice and excised tumors. (**C**) Tumor volumes of mice in each group. (**D**) Body weights of mice from each group at the end of the observation period. (**E**) The weights of excised tumors from each group. The data were presented as the mean ± SEM *P<0.05, **P<0.01, signiﬁcantly different compared with the control by test.

1. **General methods of chemistry section**

Reagents and solvents were purchased from commercial sources and were used without further purification. Melting points were determined on an X-5 micromelting apparatus, and ^1^H NMR and ^13^C NMR spectra were recorded on a Bruker 400 and 100 MHz spectrometer, respectively. High-resolution mass spectra were recorded on a Waters Micromass Q-T of Micromass spectrometer. The purity of all biologically evaluated compounds was determined to be > 95% by reverse-phase high-performance liquid chromatography (HPLC) analysis. The signal was monitored at 290 nm with a UV dector. A flow rate of 1.0 mL/min was used with amobile phase of CH_3_CN in H_2_O (60:40, v/v).

**General Method for** **(Azidophenyl)methan-1-ol (4-5) Preparation.** Arylamine (1.55 g, 1eq) was taken up in 2~4 N H_2_SO_4_ (15 mL) at 0 °C to afford a red solution. NaNO_2_ (1.30 g, 1.5 eq) was added as a solution in water (10 mL) open to the air. The reaction mixture was maintained at 0 °C for 30 min. NaN_3_ (1.23 g, 1.5 eq) in water (10 mL) was added slowly. The reaction mixture was stirred at room temperature for 1.5 h, resulting in a brownish-white precipitation. The reaction mixture was extracted with CH_2_Cl_2_, and the organic layers were dried with MgSO_4_ and concentrated under reduced pressure. Flash chromatography with EtOAc/hexane (1/4) afforded of a yellow oil.

**General Method for Azidobenzaldehyde** **(6-7) Preparation.** To a solution alcohol (2 g, 1 eq) in CH_2_Cl_2_ (40 mL) was added PCC (2 eq) in one portion. The resulting mixture was stirred at room temperature for 2.5 h. The reaction mixture was then filtered over a pad of silica gel on a fritted funnel. The solvent was removed, affording of a yellow liquid.

**General Method for Imine (8-11) Preparation.** The appropriate amine (10 mmol) was refluxed with the appropriate aldehyde (10 mmol) in ethanol (50 mL) until complete as indicated by TLC. The reaction mixture was reduced in vacuo until solid product crystallized from solution. The resulting imine was recrystallized from ethanol.

**General Method for *β*-Lactam (12-28) Synthesis.** A solution of the appropriate imine (1 mmol) and arylacetyl chloride (1 mmol) in CH_2_Cl_2_ (10 mL) under nitrogen was stirred. Triethylamine (1 mmol) was added dropwise, and the mixture was left to stir until complete as indicated by TLC. The mixture was washed with waterand then with saturated aqueous sodium bicarbonate solution. The organic layer was dried by filtration through anhydrous sodium sulfate. The organic layer was purified by flash column chromatography over silica gel (hexane/ethyl acetate = 9:1).

**General Method for *β*-Lactam-1,2,3-triazoles (29-32) Formation.** In a round-bottom flask equipped with a magnetic stirred bar, alkyne derivatives (5 mmol), azide derivatives **17** or **18** (5.5 mmol), CuSO_4_·5H_2_O (0.25 mmol), sodium ascorbate (0.5 mmol), THF (20mL), and H_2_O (20 mL) were added. The resulting mixture was stirred at room temperature for about 5 h. After thereaction, water was added and the reaction mixture was extracted with EtOAc. The combined organic layer was washed with brine, dried over anhydrous Na_2_SO_4_, and concentrated under vacuum to afford the crude products. The crude products were purified by flash column chromatography over silica gel (hexane/ethyl acetate = 4:1) to afford **29−32**.

**General Procedure for the Synthesis of *β*-lactam-triflones** **35-36.** Compounds **33-34** were prepared according to the reported method[^3^](#_ENREF_1). Diazo-triflones **34** (0.4 mmol, 2.0 equiv) was placed to a flame-dried 50 mL two-neck flask under N_2_ atmosphere. Dry toluene (10 mL) was added to the flask, and the reaction mixture was stirred at 110 °C for 1 h. Then imines(0.2 mmol, 1.0 equiv) in toluene (5 mL) was added slowly to the mixture, and the mixture was stirred at 100 °C until imine was consumed (usually 2.5 h, monitored by TLC). Removal of the solvent under reduced pressure gave the crude product, which was purified by column chromatography with ethyl acetate/hexane (1/5) as eluent toafford **35-36**.

1. **^1^H and ^13^C NMR Spectra of all β-lactam-azide derivatives**

*trans*-4-(4-azidophenyl)-1-(3,4-dimethoxyphenyl)-3-phenylazetidin-2-

one (**12**)

Yellow oil, yield: 61 %. ^1^H NMR (400 MHz, CDCl_3_) δ 7.35 – 7.20 (m, 8H), 6.98 (d, *J* = 8.5 Hz, 2H), 6.61 (d, *J* = 8.6 Hz, 1H), 6.39 (dd, *J* = 8.6, 2.3 Hz, 1H), 4.81 (d, *J* = 2.4 Hz, 1H), 4.16 (d, *J* = 2.4 Hz, 1H), 3.75 (s, 3H), 3.72 (s, 3H). ^13^C NMR (100 MHz, CDCl_3_) δ 165.01, 149.47, 145.84, 140.54, 134.62, 134.24, 131.25, 129.11, 128.02, 127.52, 127.46, 119.93, 111.33, 108.13, 102.37, 65.20, 63.45, 56.09, 55.96. HRMS (ESI): calcd C_23_H_21_N_4_O_3_, [M + H]^+^ m/z, 401.1619; found, 401.1614.
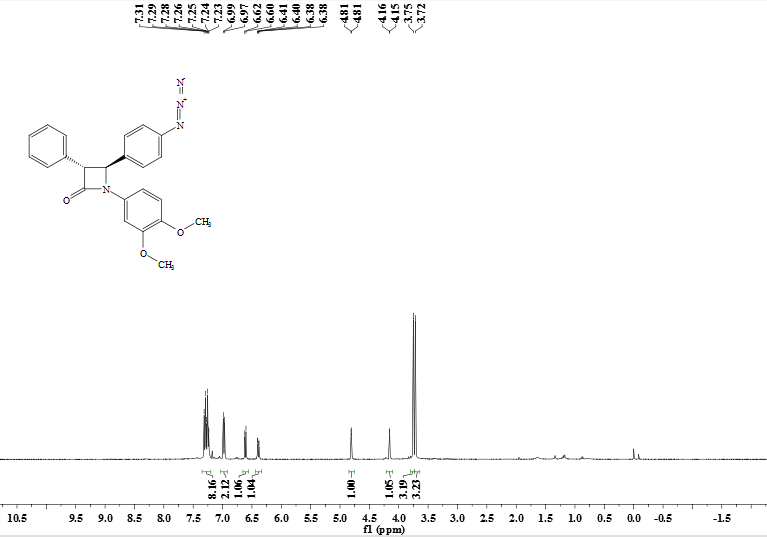


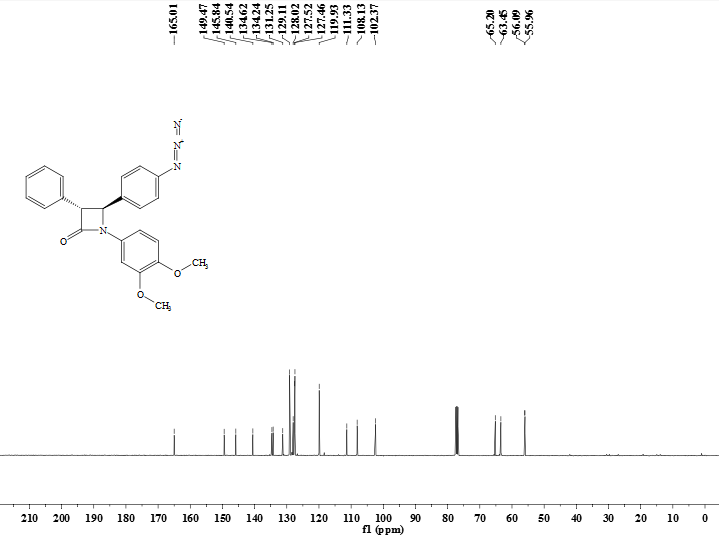


*trans* -4-(4-azidophenyl)-1-(3,4-dimethoxyphenyl)-3-(4-methoxyphenyl)

azetidin-2-one (**13**)

Brown soild, yield: 43.4 %, m.p: 130~132 ^o^C. ^1^H NMR (400 MHz, CDCl_3_) δ 7.37 (d, *J* = 8.5 Hz, 2H), 7.34 (d, *J* = 2.3 Hz, 1H), 7.24 (d, *J* = 8.7 Hz, 2H), 7.06 (d, *J* = 8.5 Hz, 2H), 6.91 (d, *J* = 8.7 Hz, 2H), 6.70 (d, *J* = 8.6 Hz, 1H), 6.46 (dd, *J* = 8.6, 2.4 Hz, 1H), 4.83 (d, *J* = 2.4 Hz, 1H), 4.18 (d, *J* = 2.3 Hz, 1H), 3.84 (s, 3H), 3.81 (s, 6H). ^13^C NMR (100 MHz, CDCl_3_) δ 165.44, 159.38, 149.47, 145.81, 140.48, 134.31, 131.29, 130.39, 128.62, 127.45, 119.90, 114.51, 111.33, 108.11, 102.39, 64.71, 63.85, 56.10, 55.96, 55.36. HRMS (ESI): calcd C_24_H_23_N_4_O_4_, [M + H]^+^ m/z, 431.1709; found, 431.1719.


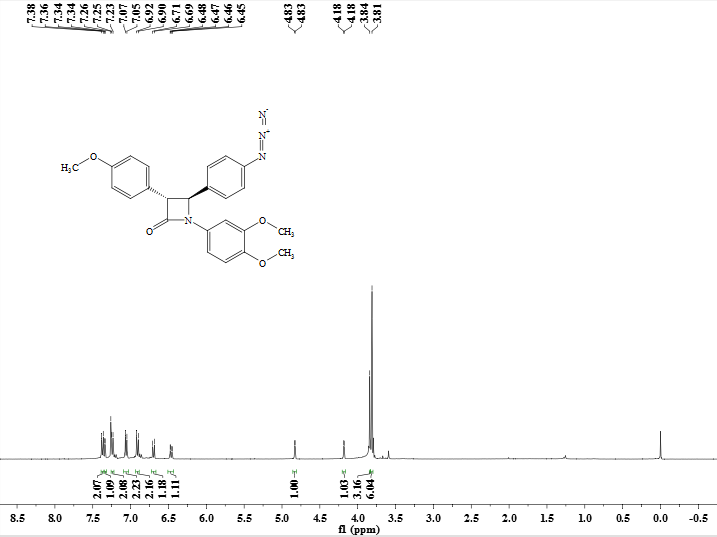


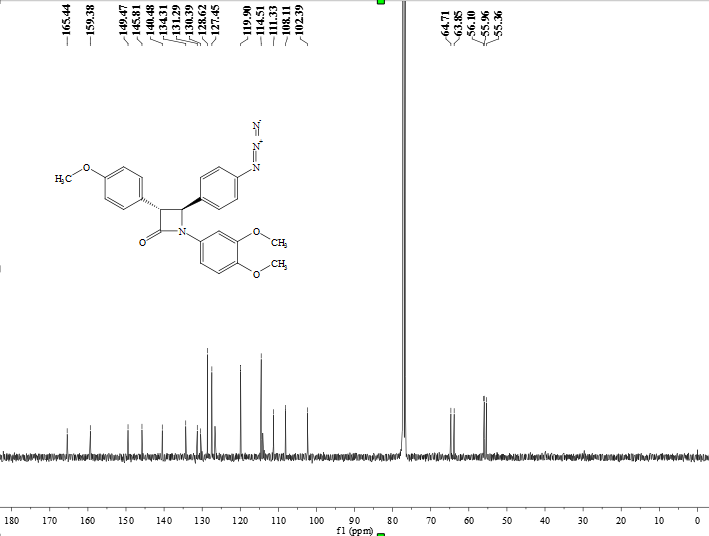


*trans*-4-(4-azidophenyl)-3-(4-chlorophenyl)-1-(4-methoxyphenyl)azetidin

-2-one **(14)**

Brown soild, yield: 25.4 %, m.p: 147~149 ^o^C. ^1^H NMR (400 MHz, CDCl_3_) δ 7.40 – 7.33 (m, 4H), 7.29 – 7.24 (m, 4H), 7.07 (t, *J* = 5.5 Hz, 2H), 6.84 – 6.77 (m, 2H), 4.84 (d, *J* = 2.4 Hz, 1H), 4.20 (d, *J* = 2.3 Hz, 1H), 3.76 (s, 3H). ^13^C NMR (100 MHz, CDCl_3_) δ 164.30, 156.37, 140.70, 133.94, 133.91, 133.10, 130.68, 129.28, 128.82, 127.47, 119.99, 118.53, 114.46, 64.51, 63.29, 55.48. HRMS (ESI): calcd C_22_H_17_ClN_4_NaO_2_, [M + Na]^+^ m/z, 427.0940; found, 427.0938.


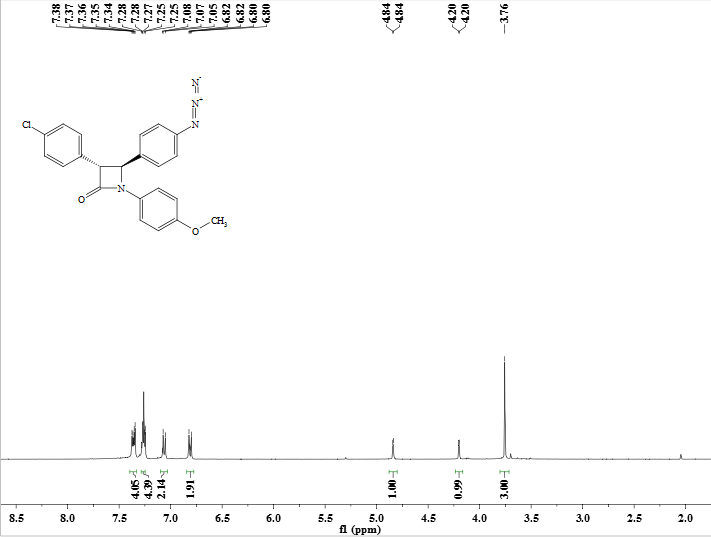


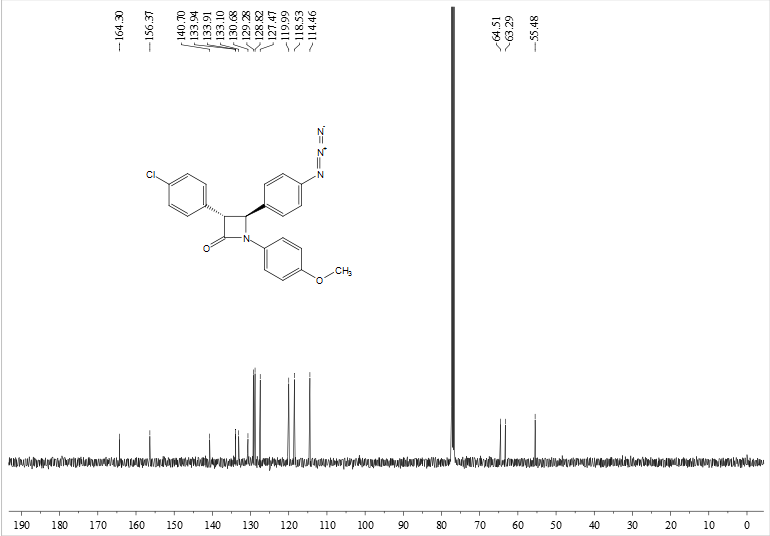


*trans*-4-(4-azidophenyl)-1-(4-methoxyphenyl)-3-phenylazetidin-2-one **(15)**

Yellow soild, yield: 45.7 %, m.p: 114~116 ^o^C. ^1^H NMR (400 MHz, CDCl_3_) δ 7.42 – 7.38 (m, 2H), 7.37 (s, 2H), 7.34 (s, 2H), 7.32 (s, 1H), 7.26 (d, *J* = 1.6 Hz, 2H), 7.06 (d, *J* = 8.5 Hz, 2H), 6.81 (d, *J* = 9.0 Hz, 2H), 4.89 (d, *J* = 2.4 Hz, 1H), 4.23 (d, *J* = 2.4 Hz, 1H), 3.75 (s, 3H). ^13^C NMR (100 MHz, CDCl_3_) δ 164.85, 156.26, 140.51, 134.65, 134.26, 130.85, 129.09, 127.98, 127.50, 127.46, 119.92, 118.50, 114.42, 65.26, 63.31, 55.47. HRMS (ESI): calcd C_22_H_19_N_4_O_2_, [M + H]^+^ m/z, 371.1509; found, 371.1508.


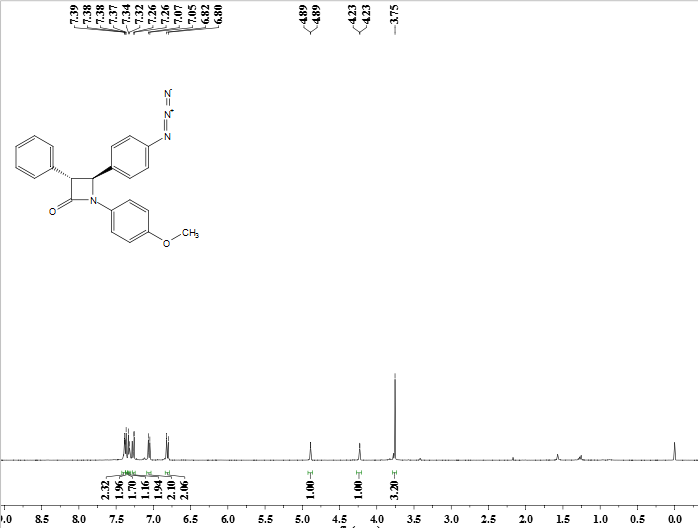


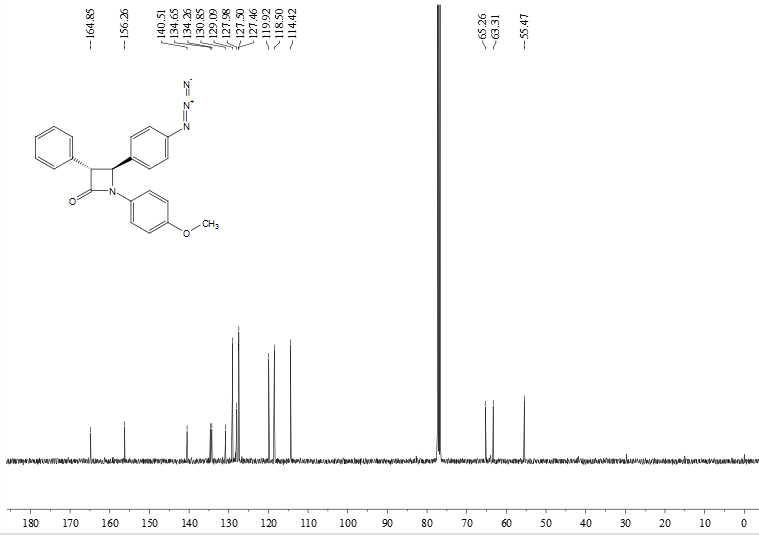


*trans*-4-(4-azidophenyl)-1-(3,4-dimethoxyphenyl)-3-(3-methoxyphenyl)

azetidin-2-one **(16)**

Brown soild, yield: 65.8 %, m.p: 150~152 ^o^C. ^1^H NMR (400 MHz, CDCl_3_) δ 7.38 (d, *J* = 8.5 Hz, 2H), 7.33 (d, *J* = 2.3 Hz, 1H), 7.28 (dd, *J* = 11.0, 4.7 Hz, 1H), 7.05 (d, *J* = 8.5 Hz, 2H), 6.91 (d, *J* = 7.6 Hz, 1H), 6.89 – 6.81 (m, 2H), 6.69 (d, *J* = 8.6 Hz, 1H), 6.48 (dd, *J* = 8.6, 2.4 Hz, 1H), 4.90 (d, *J* = 2.4 Hz, 1H), 4.21 (d, *J* = 2.3 Hz, 1H), 3.83 (s, 3H), 3.79 (d, *J* = 2.6 Hz, 6H). ^13^C NMR (100 MHz, CDCl_3_) δ 164.84, 160.11, 149.47, 145.85, 140.52, 136.04, 134.23, 131.22, 130.15, 127.54, 119.92, 119.63, 113.33, 113.24, 111.39, 108.21, 102.39, 65.08, 63.29, 56.08, 55.93, 55.29. HRMS (ESI): calcd C_24_H_23_N_4_O_4_, [M + H]^+^ m/z, 431.1710; found, 431.1719.


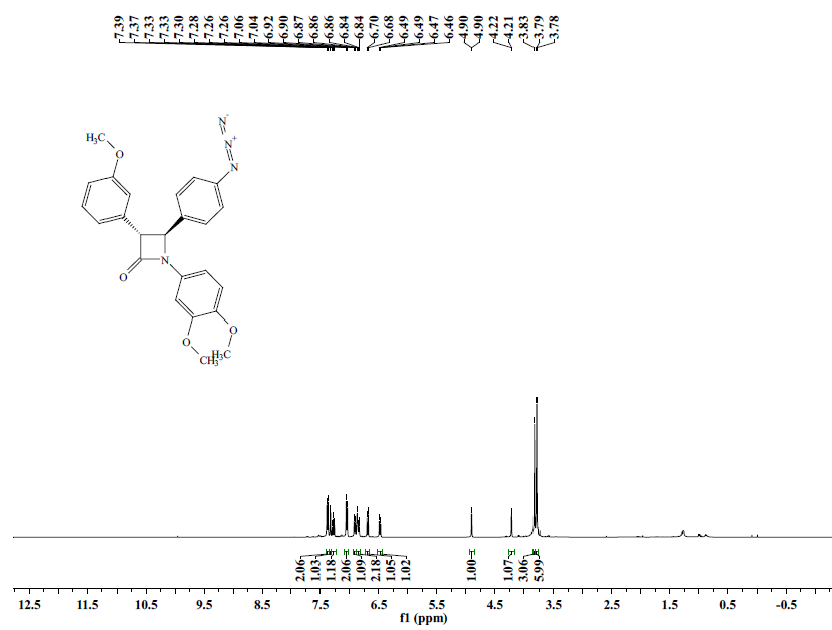


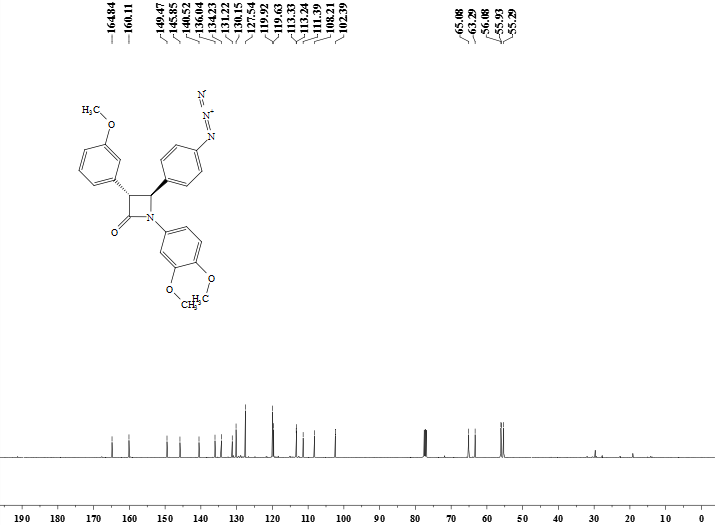


*trans*-4-(4-azidophenyl)-3-(4-methoxyphenyl)-1-(3,4,5-trimethoxyphenyl)

azetidin-2-one **(17)**

Yellow soild, yield: 46.2 %, m.p: 107~109 ^o^C. ^1^H NMR (400 MHz, CDCl_3_) δ 7.39 (d, *J* = 8.5 Hz, 2H), 7.24 (d, *J* = 8.6 Hz, 2H), 7.07 (d, *J* = 8.5 Hz, 2H), 6.91 (d, *J* = 8.7 Hz, 2H), 6.58 (s, 2H), 4.84 (d, *J* = 2.5 Hz, 1H), 4.20 (d, *J* = 2.4 Hz, 1H), 3.81 (s, 3H), 3.78 (s, 3H), 3.72 (s, 6H). ^13^C NMR (100 MHz, CDCl_3_) δ 165.77, 159.41, 153.61, 140.58, 134.67, 134.21, 133.55, 128.61, 127.47, 126.48, 119.93, 114.52, 94.87, 64.62, 63.95, 60.97, 56.08, 55.36. HRMS (ESI): calcd C_25_H_25_N_4_O_5_, [M + H]^+^ m/z,461.1816;found,461.1825.
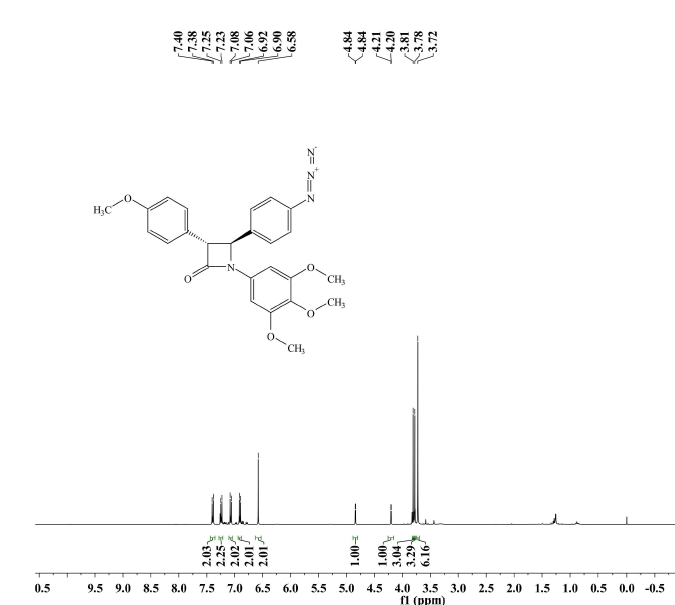


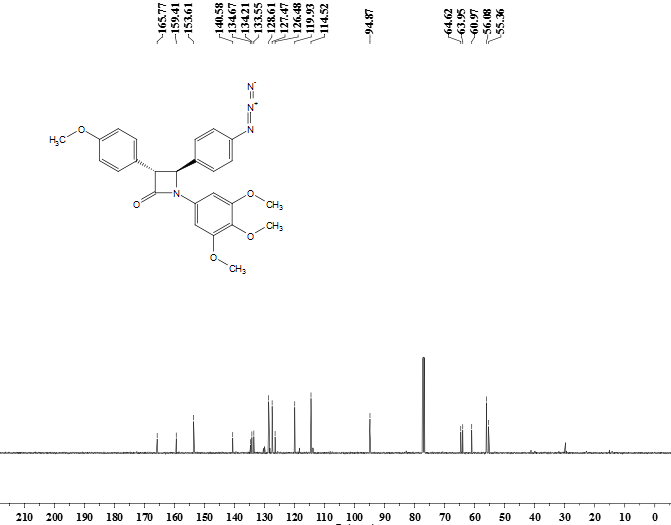


*trans*-4-(4-azidophenyl)-3-phenyl-1-(3,4,5-trimethoxyphenyl)azetidin-2-

one **(18)**

Yellow soild, yield: 37.6 %, m.p: 66~68 ^o^C.. ^1^H NMR (400 MHz, CDCl_3_) δ 7.37 (d, *J* = 24.2 Hz, 7H), 7.09 (s, 2H), 6.58 (s, 2H), 4.90 (s, 1H), 4.26 (s, 1H), 3.75 (d, *J* = 20.4 Hz, 9H). ^13^C NMR (100 MHz, CDCl_3_) δ 165.30, 153.63, 140.67, 134.73, 134.46, 134.15, 133.50, 129.13, 128.08, 127.51, 127.43, 119.96, 94.88, 65.13, 63.57, 60.97, 56.09. HRMS (ESI): calcd C_24_H_23_N_4_O_4_, [M + H]^+^ m/z, 431.1715; found, 431.1719.


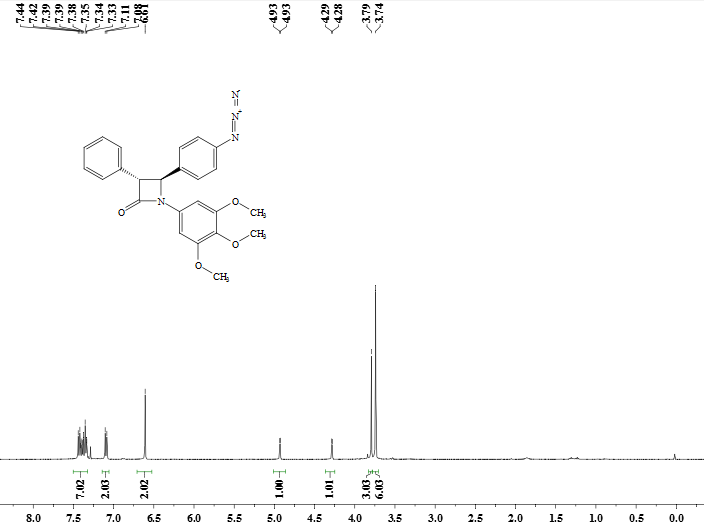


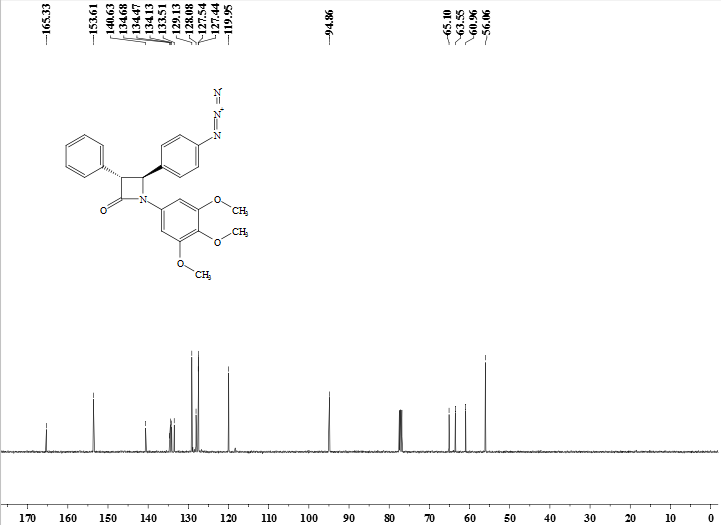


*trans*-4-(4-azidophenyl)-3-(3-methoxyphenyl)-1-(3,4,5-trimethoxyphenyl)

azetidin-2-one (**19**)

Brown soild, yield: 66.3 %, m.p: 64~66 ^o^C. ^1^H NMR (400 MHz, CDCl_3_) δ 7.33 (d, *J* = 8.4 Hz, 2H), 7.22 (dd, *J* = 8.7, 7.8 Hz, 1H), 7.00 (d, *J* = 8.4 Hz, 2H), 6.84 (d, *J* = 7.7 Hz, 1H), 6.81 – 6.75 (m, 2H), 6.50 (s, 2H), 4.82 (d, *J* = 2.4 Hz, 1H), 4.16 (d, *J* = 2.4 Hz, 1H), 3.73 (s, 3H), 3.70 (s, 3H), 3.65 (s, 6H). ^13^C NMR (100 MHz, CDCl_3_) δ 165.14, 160.12, 153.60, 140.66, 135.87, 134.68, 134.10, 133.48, 130.20, 127.52, 119.95, 119.62, 113.37, 113.23, 94.86, 65.02, 63.44, 60.98, 56.08, 55.34. HRMS (ESI): calcd C_25_H_25_N_4_O_5_, [M + H]^+^ m/z, 483.1816; found, 461.1825.


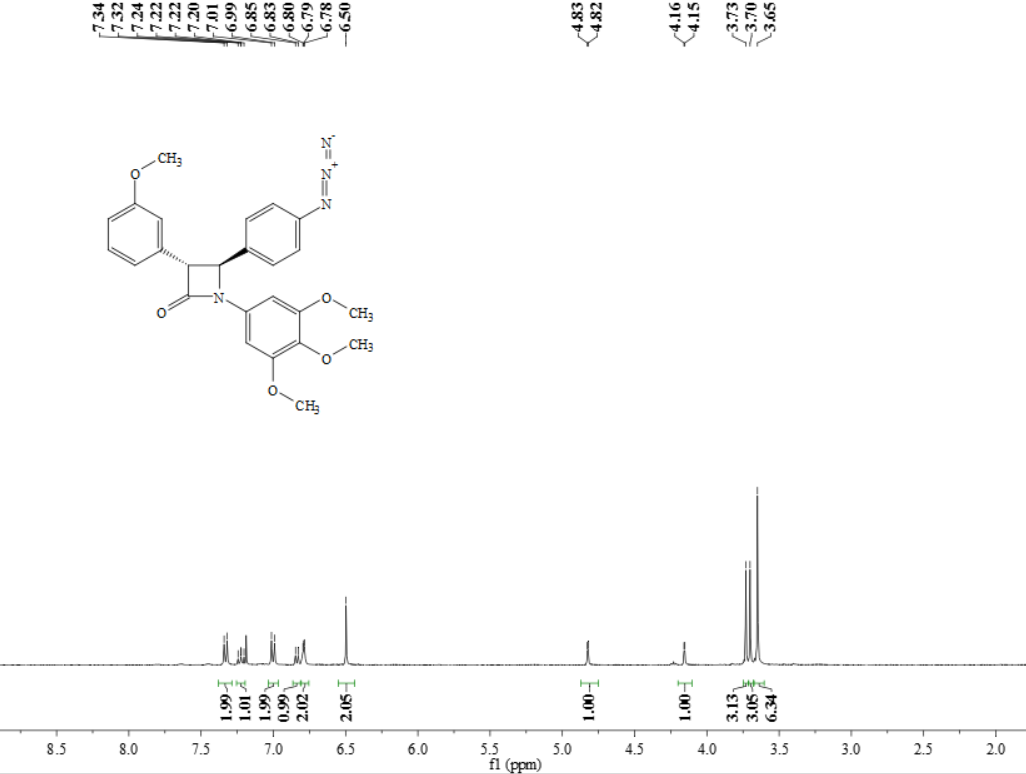


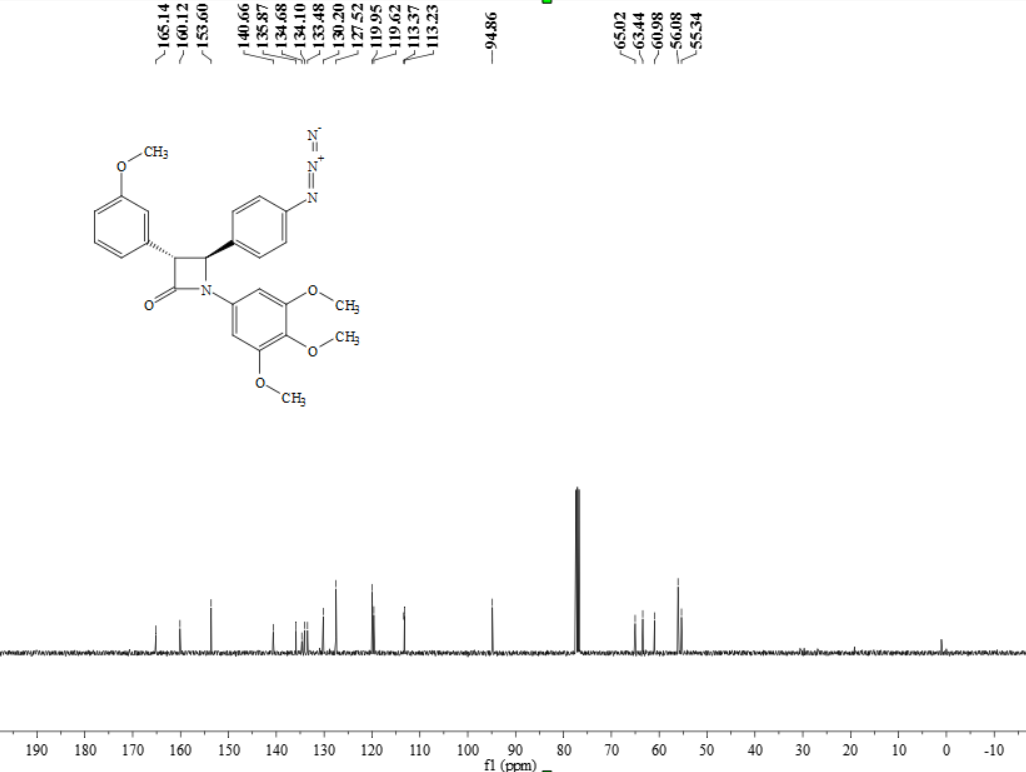


*trans*-4-(4-azidophenyl)-3-(4-chlorophenyl)-1-(3,4,5-trimethoxyphenyl)

azetidin-2-one **(20)**

Brown soild, yield: 35.5 %, m.p: 147~149 ^o^C. ^1^H NMR (400 MHz, CDCl_3_) δ 7.46 – 7.34 (m, 3H), 7.28 (s, 2H), 7.19 (d, *J* = 7.0 Hz, 1H), 7.08 (d, *J* = 8.0 Hz, 1H), 7.02 (s, 1H), 6.56 (s, 2H), 4.84 (s, 1H), 4.25 (s, 1H), 3.79 (s, 3H), 3.73 (s, 6H). ^13^C NMR (100 MHz, CDCl_3_) δ 164.63, 153.66, 141.47, 139.45, 134.91, 134.09, 133.28, 132.79, 130.92, 129.33, 128.80, 122.33, 119.41, 116.47, 94.91, 64.19, 63.54, 60.97, 56.11. HRMS (ESI): calcd C_24_H_21_ClN_4_NaO_4_, [M + Na]^+^ m/z, 487.1152; found, 487.1149.


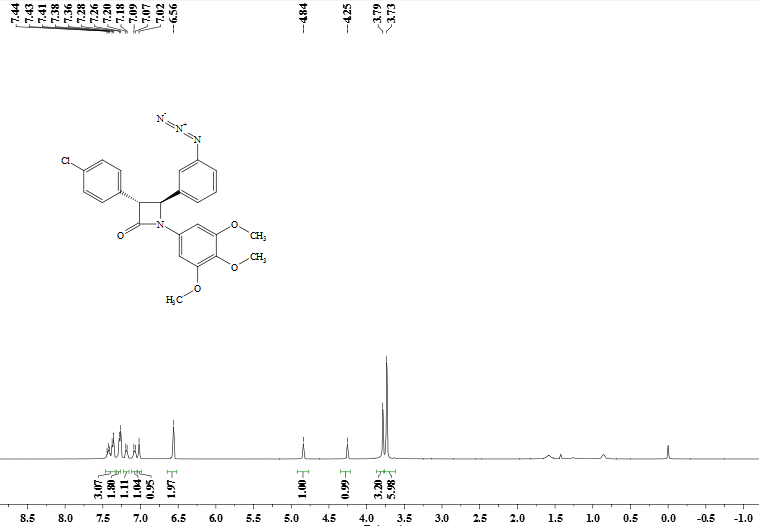


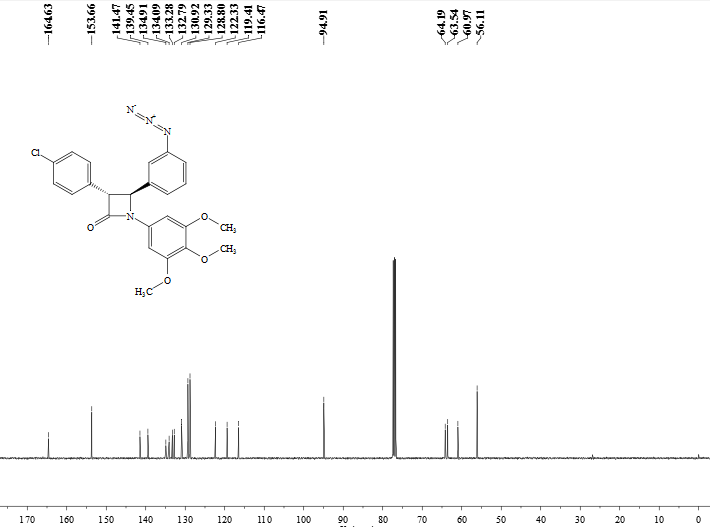


*trans*-4-(3-azidophenyl)-1-(2,4-dimethoxyphenyl)-3-(4-methoxyphenyl)

azetidin-2-one **(21)**

Yellow soild, yield: 42.3 %, m.p: 86~88 ^o^C. ^1^H NMR (400 MHz, CDCl_3_) δ 7.63 (d, *J* = 8.7 Hz, 1H), 7.35 – 7.25 (m, 3H), 7.11 (d, *J* = 7.7 Hz, 1H), 6.98 (t, *J* = 1.7 Hz, 1H), 6.92 (ddd, *J* = 8.6, 4.4, 1.7 Hz, 3H), 6.45 (dd, *J* = 8.7, 2.6 Hz, 1H), 6.40 (d, *J* = 2.5 Hz, 1H), 5.13 (d, *J* = 2.4 Hz, 1H), 4.19 (d, *J* = 2.4 Hz, 1H), 3.79 (s, 3H), 3.74 (s, 3H), 3.68 (s, 3H). ^13^C NMR (100 MHz, CDCl_3_) δ 165.96, 158.22, 157.83, 152.34, 140.31, 139.64, 129.26, 127.72, 126.13, 124.46, 121.48, 117.65, 117.14, 115.56, 113.38, 103.49, 98.61, 65.86, 63.98, 54.46, 54.28. HRMS (ESI): calcd C_24_H_23_N_4_O_4_, [M + Na]^+^ m/z, 431.1715; found, 431.1719.


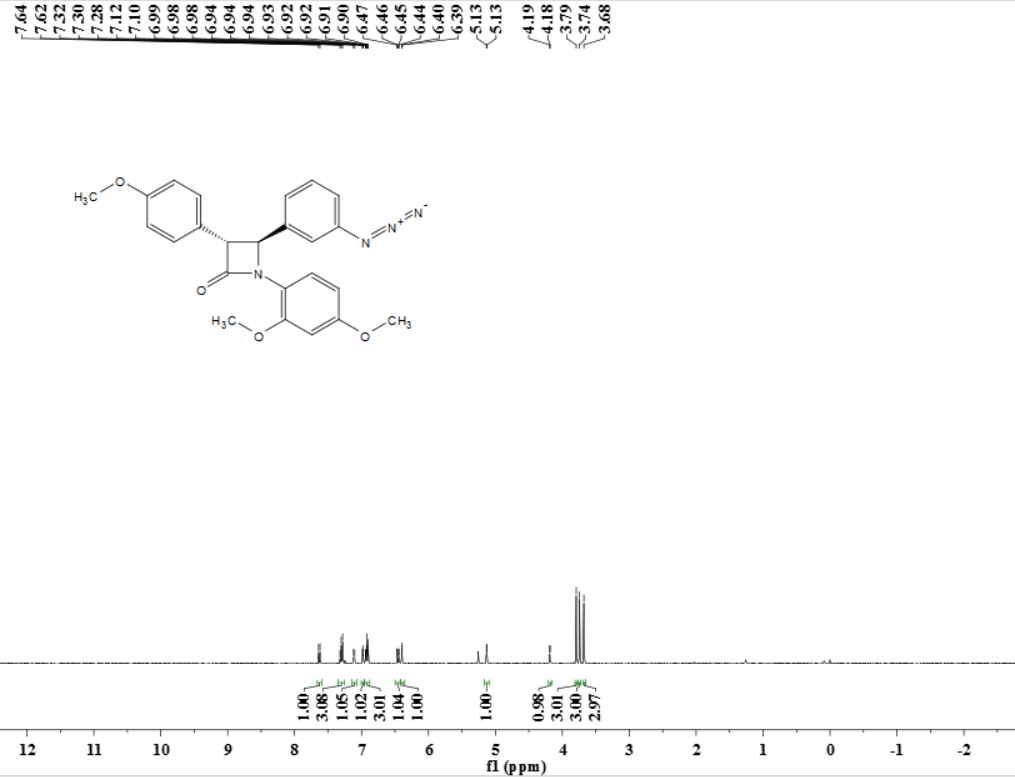


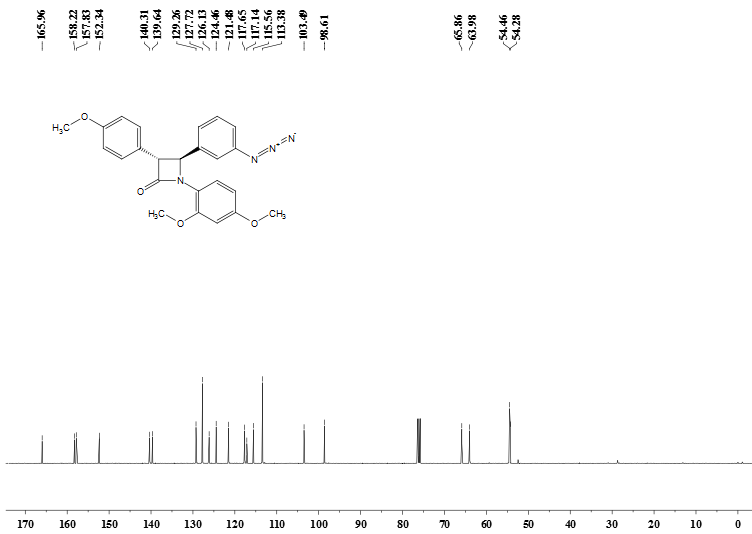


*trans*-4-(3-azidophenyl)-1-(2,4-dimethoxyphenyl)-3-(4-fluorophenyl)azet

idin-2-one **(22)**

Brown soild, yield: 27.3 %, m.p: 90~92 ^o^C. ^1^H NMR (400 MHz, CDCl_3_) δ 7.54 (d, *J* = 8.7 Hz, 1H), 7.26 (ddd, *J* = 15.6, 8.8, 4.9 Hz, 3H), 7.05 – 6.95 (m, 3H), 6.91 – 6.84 (m, 2H), 6.38 (dd, *J* = 8.7, 2.6 Hz, 1H), 6.32 (d, *J* = 2.5 Hz, 1H), 5.05 (d, *J* = 2.4 Hz, 1H), 4.13 (d, *J* = 2.4 Hz, 1H), 3.67 (s, 3H), 3.61 (s, 3H). ^13^C NMR (100 MHz, CDCl_3_) δ 165.38, 162.56, 160.11, 157.95, 152.35, 139.99, 129.86, 129.34, 128.28, 124.51, 121.47, 117.79, 116.94, 115.58, 114.99, 103.52, 98.62, 65.65, 63.69, 54.47. HRMS (ESI): calcd C_23_H_20_FN_4_O_3_, [M + H]^+^ m/z, 419.1511; found, 419.1519.


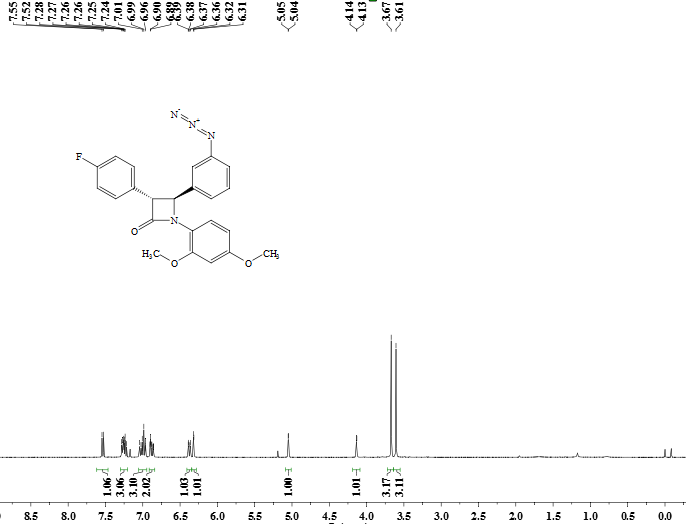


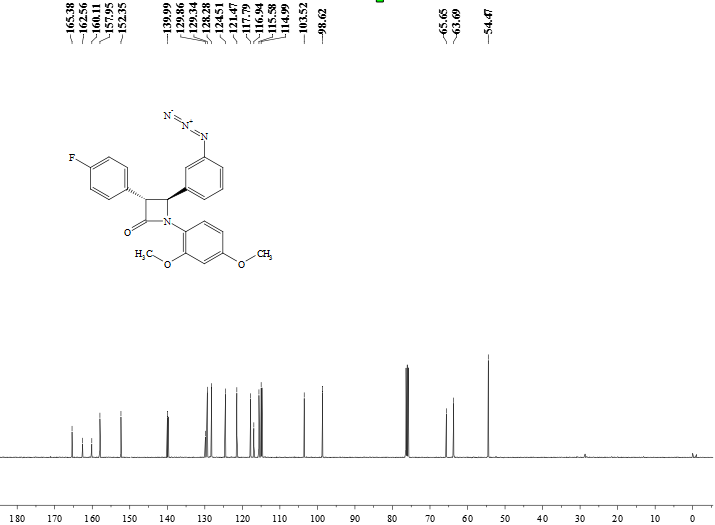


*trans*-4-(3-azidophenyl)-3-(3-methoxyphenyl)-1-(3,4,5-trimethoxyphenyl)

azetidin-2-one **(23)**

White soild, yield: 32.9 %, m.p: 164~166 ^o^C. ^1^H NMR (400 MHz, CDCl_3_) δ 7.34 (t, *J* = 7.8 Hz, 1H), 7.23 (dd, *J* = 8.9, 7.7 Hz, 1H), 7.12 (d, *J* = 7.8 Hz, 1H), 7.03 – 6.94 (m, 2H), 6.89 – 6.77 (m, 3H), 6.50 (s, 2H), 4.81 (d, *J* = 2.5 Hz, 1H), 4.18 (d, *J* = 2.5 Hz, 1H), 3.73 (s, 3H), 3.71 (s, 3H), 3.66 (s, 6H). ^13^C NMR (100 MHz, CDCl_3_) δ 165.06, 160.12, 153.62, 141.35, 139.72, 135.75, 134.73, 133.43, 130.86, 130.22, 122.43, 119.62, 119.30, 116.52, 113.43, 113.23, 94.86, 64.84, 63.48, 60.98, 56.09, 55.34. HRMS (ESI): calcd C_25_H_25_N_4_O_5_ [M + H]^+^ m/z, 461.1828; found,461.1825.
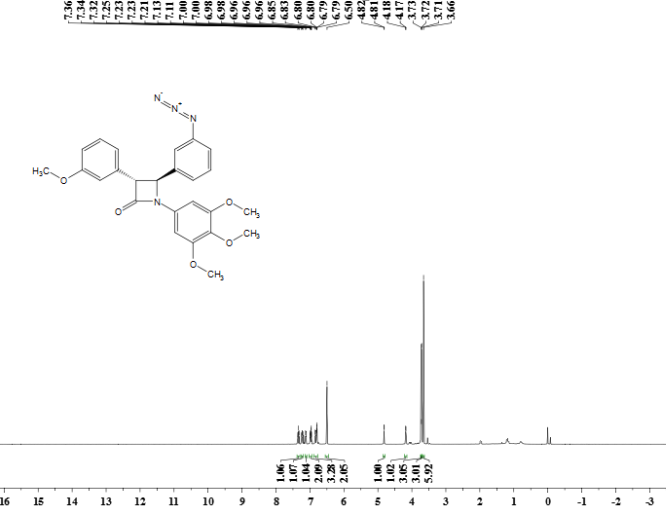


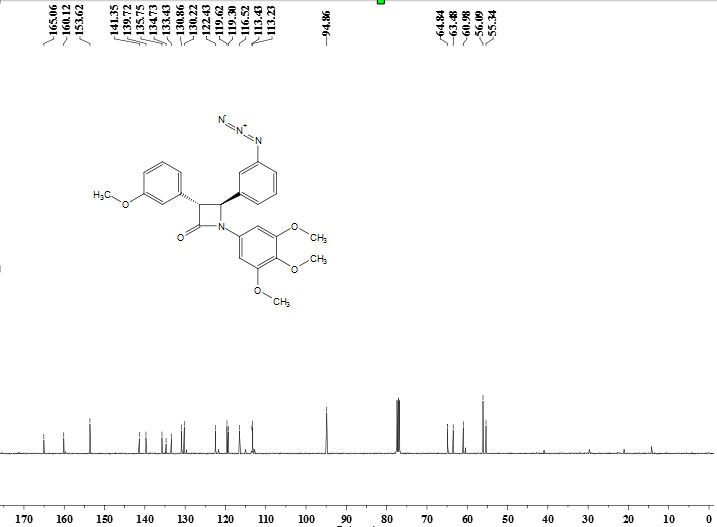


*trans*-4-(3-azidophenyl)-3-(4-fluorophenyl)-1-(3,4,5-trimethoxyphenyl)

azetidin-2-one **(24)**

Yellow soild, yield: 35.6 %, m.p: 112~114 ^o^C. ^1^H NMR (400 MHz, CDCl_3_) δ 7.35 (t, *J* = 7.8 Hz, 1H), 7.28 – 7.20 (m, 2H), 7.12 (d, *J* = 7.8 Hz, 1H), 7.05 – 6.97 (m, 3H), 6.95 (d, *J* = 1.8 Hz, 1H), 6.50 (s, 2H), 4.77 (d, *J* = 2.5 Hz, 1H), 4.19 (d, *J* = 2.4 Hz, 1H), 3.71 (s, 3H), 3.66 (s, 6H). ^13^C NMR (100 MHz, CDCl_3_) δ 163.94, 162.71, 160.25, 152.63, 140.41, 138.52, 133.85, 132.31, 129.88, 129.13, 128.15, 121.32, 118.34, 115.20, 93.89, 63.12, 62.72, 59.95, 55.08. HRMS (ESI): calcd C_24_H_21_FN_4_NaO_4_, [M + Na]^+^ m/z, 471.1443; found, 471.1445.


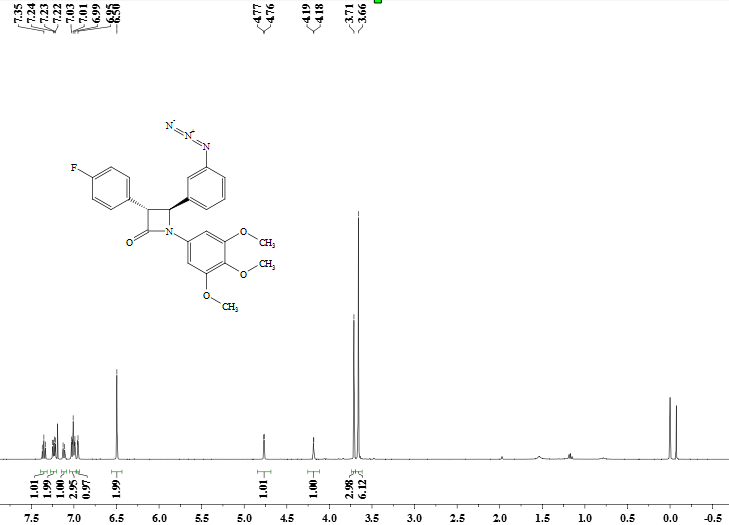


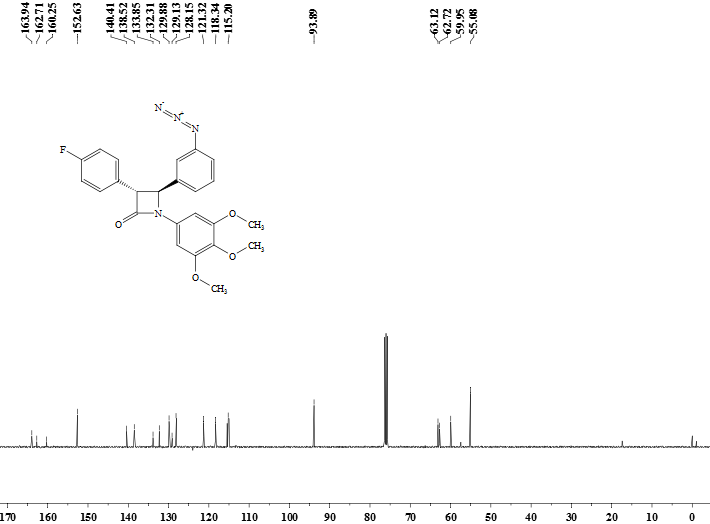


*trans*-4-(3-azidophenyl)-3-phenyl-1-(3,4,5-trimethoxyphenyl)azetidin-2-one (**25**)

White soild, yield: 48.9 %, m.p: 128~129 ^o^C. ^1^H NMR (400 MHz, CDCl_3_) δ 7.48 – 7.29 (m, 6H), 7.20 (d, *J* = 7.7 Hz, 1H), 7.12 – 7.01 (m, 2H), 6.58 (s, 2H), 4.89 (d, *J* = 2.5 Hz, 1H), 4.28 (d, *J* = 2.5 Hz, 1H), 3.79 (s, 3H), 3.73 (s, 6H). ^13^C NMR (100 MHz, CDCl_3_) δ 165.18, 153.63, 141.35, 139.76, 134.77, 134.34, 133.44, 130.84, 129.14, 128.12, 127.43, 122.39, 119.27, 116.52, 94.87, 64.94, 63.60, 60.97, 56.09. HRMS (ESI): calcd C_24_H_23_N_4_O_4_, [M + H]^+^ m/z, 431.1716; found,431.1719.
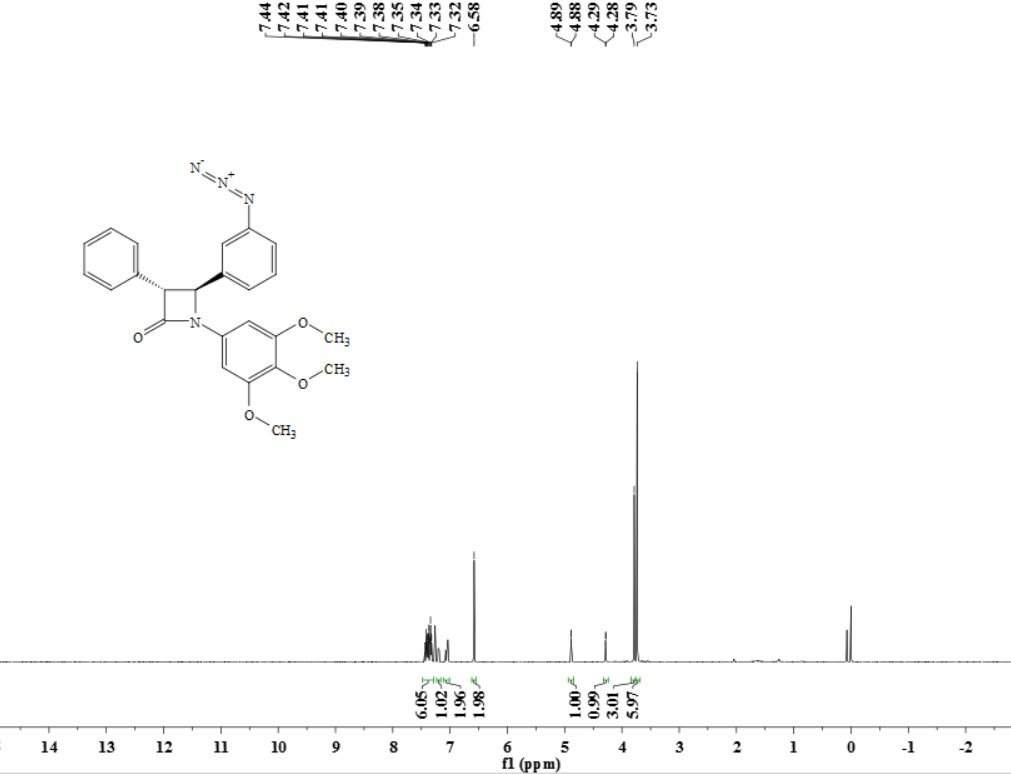


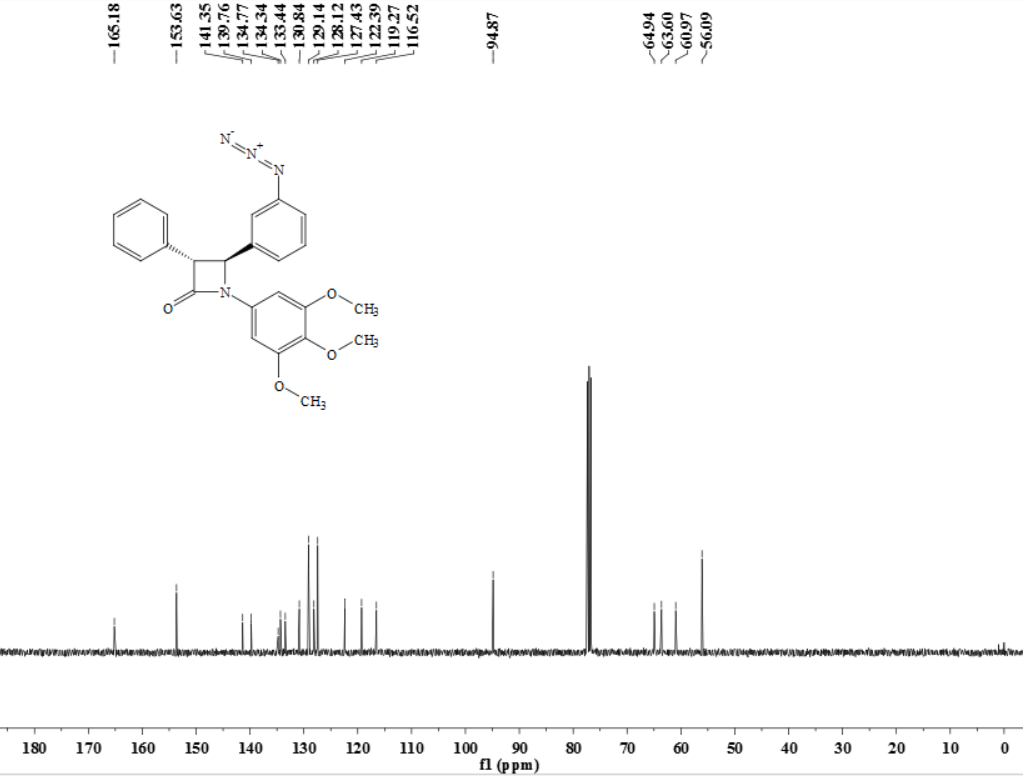


*trans*-4-(4-azidophenyl)-3-(thiophen-2-yl)-1-(3,4,5-trimethoxyphenyl)

azetidin-2-one **(26)**

Brown soild, yield: 10.3 %, m.p: 122~124 ^o^C. ^1^H NMR (400 MHz, CDCl_3_) δ 7.45 (d, *J* = 8.2 Hz, 2H), 7.36 – 7.27 (m, 1H), 7.07 (dd, *J* = 15.1, 10.0 Hz, 4H), 6.59 (d, *J* = 6.4 Hz, 2H), 4.97 (d, *J* = 3.9 Hz, 1H), 4.49 (d, *J* = 4.1 Hz, 1H), 3.78 (dd, *J* = 20.8, 6.4 Hz, 9H). ^13^C NMR (100 MHz, CDCl_3_) δ 164.11, 153.64, 140.80, 135.81, 135.80, 134.82, 133.56, 133.44, 127.46, 125.91, 125.53, 119.99, 94.96, 64.30, 60.97, 60.24, 56.10. HRMS (ESI): calcd C_22_H_20_N_4_NaO_4_S, [M + Na]^+^ m/z, 459.1104; found, 459.1103.


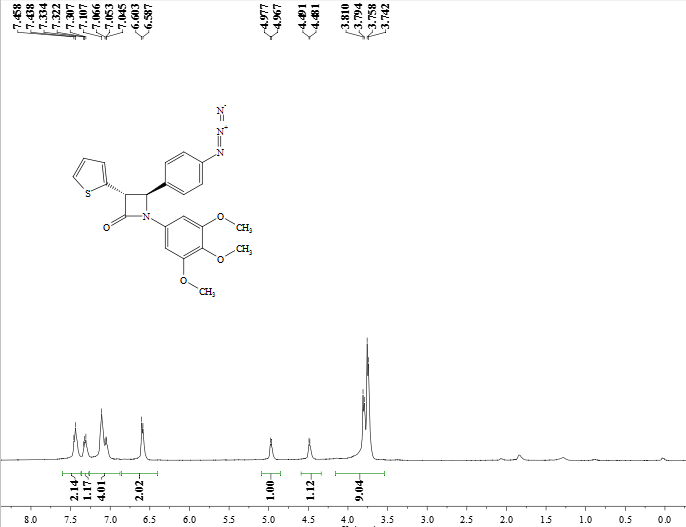


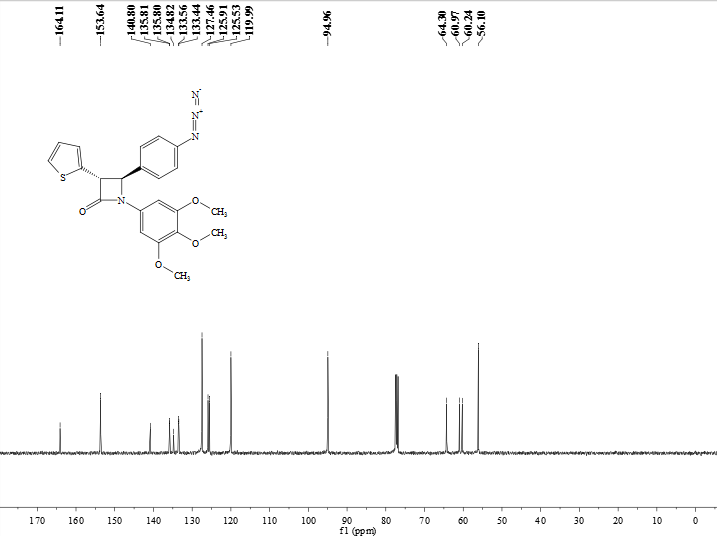


*trans*-4-phenyl-3-(thiophen-2-yl)-1-(3,4,5-trimethoxyphenyl)azetidin-2-one **(27)**

Brown soild, yield: 25.3 %, m.p: 58~60 ^o^C. ^1^H NMR (400 MHz, CDCl_3_) δ 7.30 (d, *J* = 8.5 Hz, 2H), 7.22 – 7.15 (m, 3H), 6.96 (ddd, *J* = 15.8, 5.3, 3.5 Hz, 4H), 6.76 – 6.68 (m, 2H), 4.85 (d, *J* = 2.5 Hz, 1H), 4.34 (d, *J* = 2.2 Hz, 1H), 3.67 (s, 3H). ^13^C NMR (100 MHz, CDCl_3_) δ 163.64, 156.37, 140.67, 136.07, 133.69, 130.77, 127.44 (d, *J* = 4.8 Hz), 125.84, 125.41, 119.96, 118.60, 114.45, 64.07, 60.36, 55.48. HRMS (ESI): calcd C_20_H_17_N_4_O_2_S, [M + H]^+^ m/z, 377.1068; found, 377.1072.


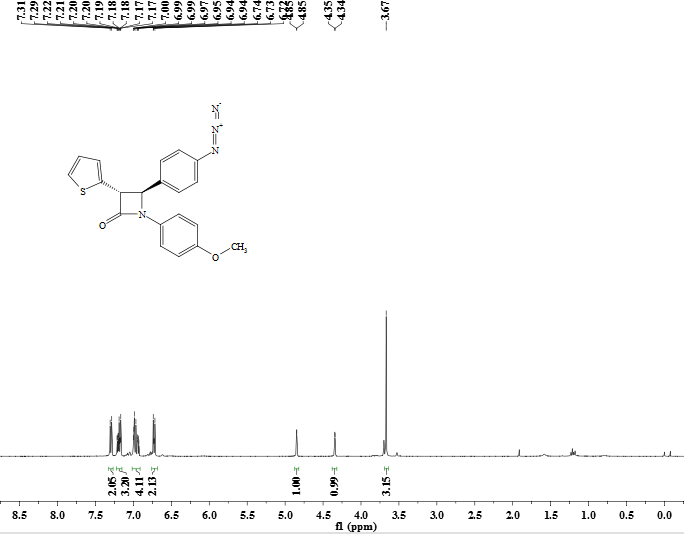


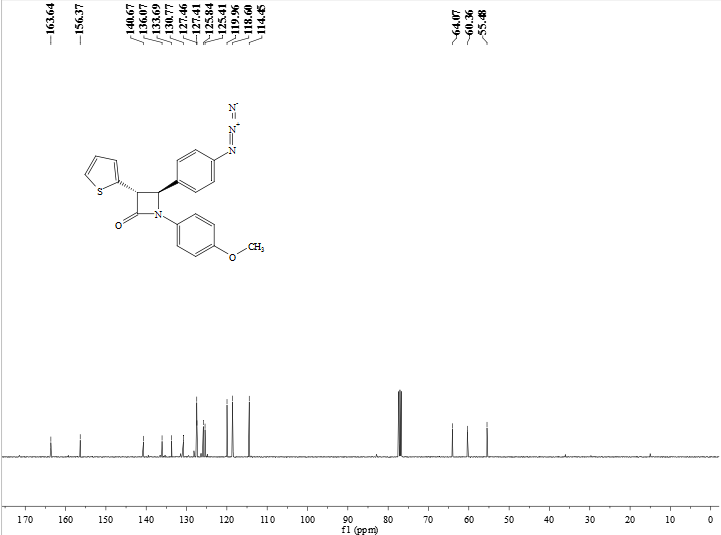


*trans*-4-(3-azidophenyl)-3-(thiophen-2-yl)-1-(3,4,5-trimethoxyphenyl)aze

tidin-2-one **(28)**

Yellow soild, yield: 26.8 %, m.p: 106~108 ^o^C. ^1^H NMR (400 MHz, CDCl_3_) δ 7.32 (t, *J* = 7.8 Hz, 1H), 7.19 (dd, *J* = 5.1, 0.9 Hz, 1H), 7.11 (d, *J* = 7.7 Hz, 1H), 6.98 (dd, *J* = 8.9, 5.7 Hz, 3H), 6.92 (dd, *J* = 5.0, 3.6 Hz, 1H), 6.48 (s, 2H), 4.85 (d, *J* = 2.5 Hz, 1H), 4.39 (d, *J* = 2.3 Hz, 1H), 3.69 (s, 3H), 3.63 (s, 6H). ^13^C NMR (100 MHz, CDCl_3_) δ 163.98, 153.64, 141.36, 139.19, 135.68, 134.85, 133.38, 130.90, 127.45, 125.97, 125.59, 122.35, 119.42, 116.47, 94.95, 64.30, 60.96, 60.07, 56.09. HRMS (ESI): calcd C_22_H_20_N_4_NaO_4_S, [M + Na]^+^ m/z, 459.1107; found, 459.1103.


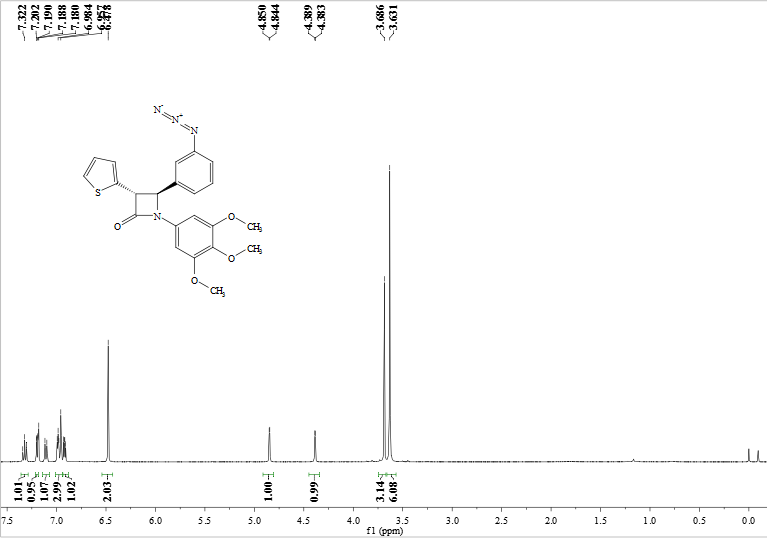


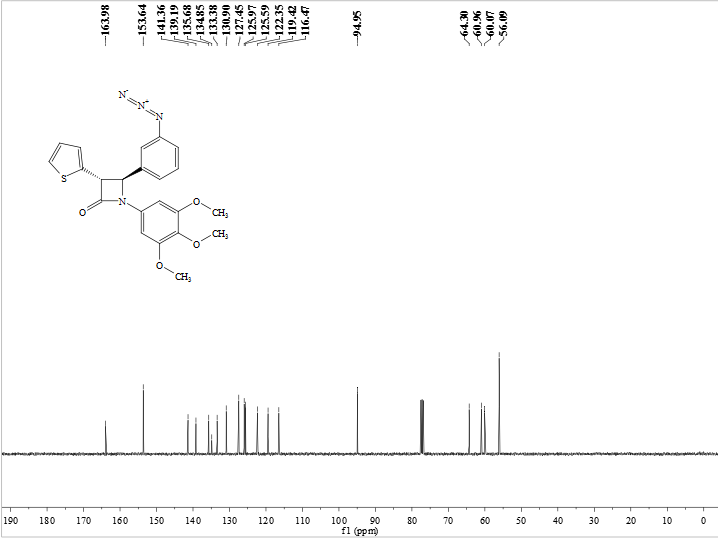


*trans*-4-(4-(4-((3-nitrophenoxy)methyl)-1H-1,2,3-triazol-1-yl)phenyl)-3-phenyl-1-(3,4,5-trimethoxyphenyl)azetidin-2-one **(29)**

White soild, yield: 78 %, m.p: 142~145 ^o^C. ^1^H NMR (400 MHz, CDCl_3_) δ 8.18 (s, 1H), 7.85 (d, *J* = 8.5 Hz, 4H), 7.61 (d, *J* = 8.5 Hz, 2H), 7.39 (dt, *J* = 16.1, 7.8 Hz, 6H), 6.62 (s, 2H), 5.37 (s, 2H), 5.03 (d, *J* = 2.4 Hz, 1H), 4.32 (d, *J* = 2.3 Hz, 1H), 3.79 (s, 3H), 3.74 (s, 6H). ^13^C NMR (100 MHz, CDCl_3_) δ 165.06, 158.59, 153.70, 149.18, 143.96, 138.63, 137.03, 134.86, 134.17, 133.33, 130.25, 129.22, 128.25, 127.48, 127.44, 121.56, 121.43, 121.20, 116.40, 109.52, 94.90, 65.19, 63.24, 62.29, 60.97, 56.12, 29.70. HRMS (ESI): calcd C_33_H_30_N_5_O_7_, [M + Na]^+^ m/z, 608.2141; found, 608.2145.


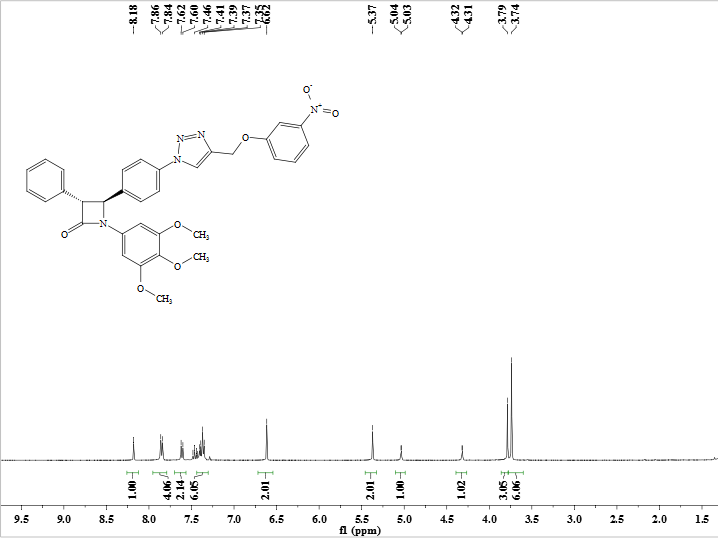


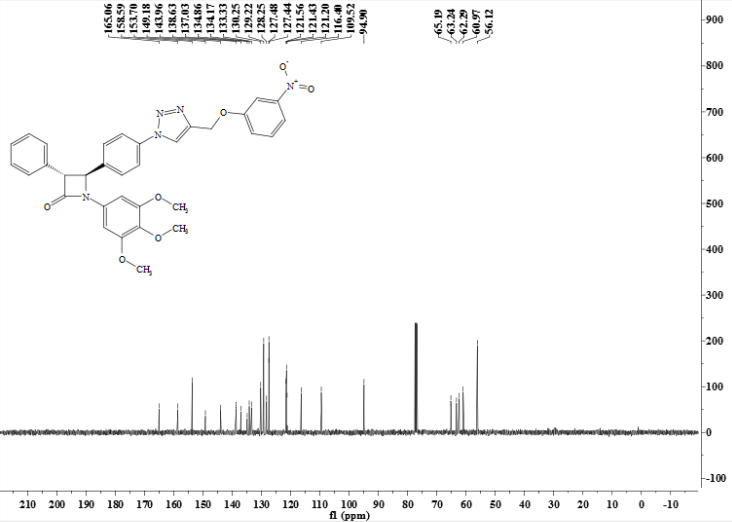


*trans*-3-(4-methoxyphenyl)-4-(4-(4-phenyl-1H-1,2,3-triazol-1-yl)phenyl)-1-(3,4,5-trimethoxyphenyl)azetidin-2-one **(30)**

White soild, yield: 88 %, m.p: 90~91 ^o^C. ^1^H NMR (400 MHz, CDCl_3_) δ 8.24 (s, 1H), 7.91 (dd, *J* = 14.2, 7.9 Hz, 4H), 7.60 (d, *J* = 8.5 Hz, 2H), 7.48 (t, *J* = 7.6 Hz, 2H), 7.40 (t, *J* = 7.3 Hz, 1H), 7.29 (d, *J* = 6.7 Hz, 2H), 6.95 (d, *J* = 8.6 Hz, 2H), 6.63 (s, 2H), 4.97 (d, *J* = 2.4 Hz, 1H), 4.27 (d, *J* = 2.4 Hz, 1H), 3.84 (s, 3H), 3.80 (s, 3H), 3.76 (s, 6H). ^13^C NMR (100 MHz, CDCl_3_) δ 165.54, 159.53, 153.71, 148.67, 138.38, 137.23, 134.82, 133.43, 130.00, 129.00, 128.66, 128.63, 127.36, 126.22, 125.89, 121.31, 117.40, 114.61, 94.88, 64.77, 63.76, 61.00, 56.14, 55.40. HRMS (ESI): calcd C_33_H_31_N_4_O_5_, [M + Na]^+^ m/z, 563.2290; found, 563.2294.


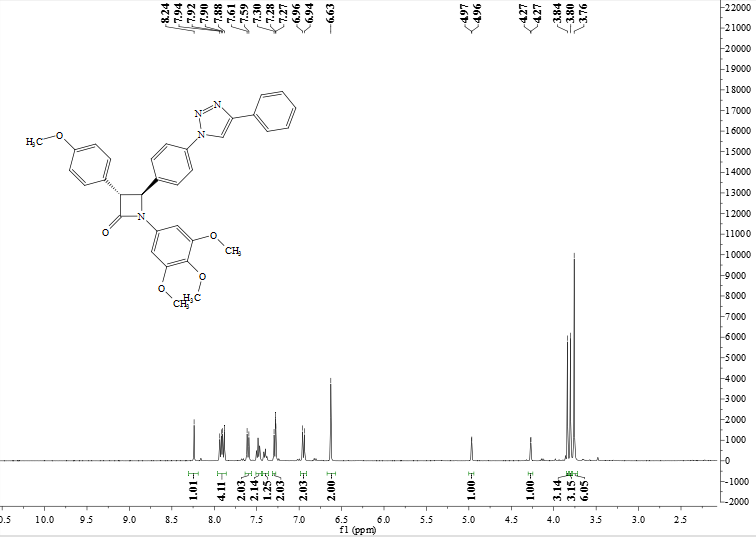


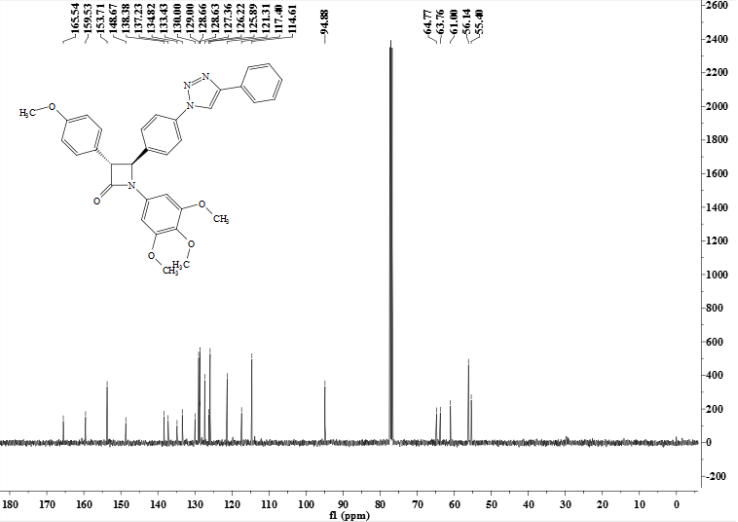


(1-(4-(*trans*-3-(4-methoxyphenyl)-4-oxo-1-(3,4,5-trimethoxyphenyl)azeti

din-2-yl)phenyl)-1H-1,2,3-triazol-4-yl)methylpyrrolidine-1-carbodithioat

e **(31)**

Gray soild, yield: 88 %, m.p: 92~94 ^o^C. ^1^H NMR (400 MHz, CDCl_3_) δ 8.23 (s, 1H), 7.81 (d, *J* = 8.5 Hz, 2H), 7.56 (d, *J* = 8.5 Hz, 2H), 7.27 (d, *J* = 9.6 Hz, 2H), 6.94 (d, *J* = 8.6 Hz, 2H), 6.60 (s, 2H), 4.94 (d, *J* = 2.4 Hz, 1H), 4.79 (s, 2H), 4.25 (d, *J* = 2.4 Hz, 1H), 3.95 (s, 2H), 3.84 (s, 3H), 3.80 (s, 3H), 3.75 (s, 6H), 3.66 (s, 2H), 2.10 – 2.05 (m, 2H), 2.03 – 1.97 (m, 2H). ^13^C NMR (100 MHz, CDCl_3_) δ 165.53, 159.50, 153.68, 138.23, 137.23, 134.77, 133.41, 129.89, 128.65, 127.24, 126.23, 121.36, 119.87, 114.59, 113.78, 94.82, 64.72, 63.75, 60.99, 56.12, 55.39, 50.70, 30.79, 29.71, 26.08, 24.30. HRMS (ESI): calcd C_33_H_36_N_5_O_5_S_2_, [M + Na]^+^ m/z, 646.2150; found, 646.2158.


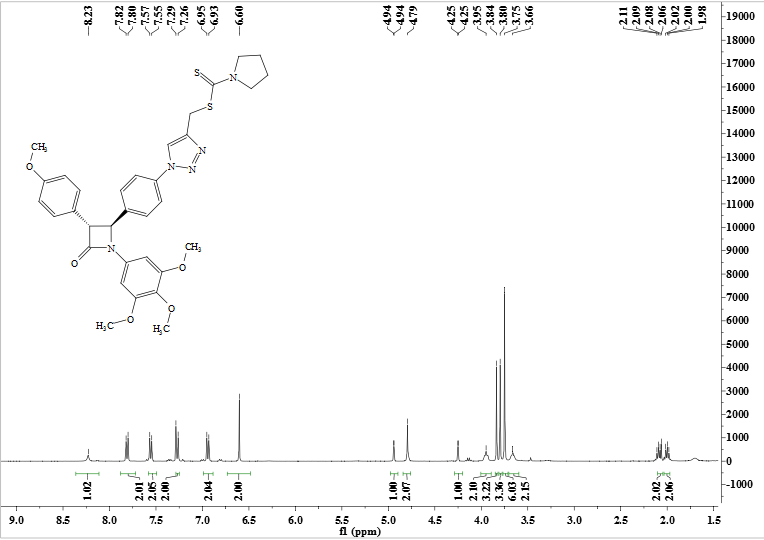


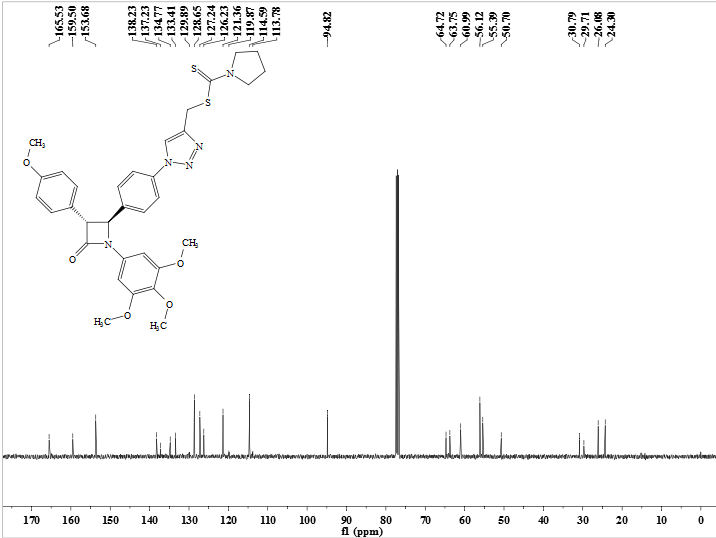


(1-(4-(*trans*-4-oxo-3-phenyl-1-(3,4,5-trimethoxyphenyl)azetidin-2-yl)phe

nyl)-1H-1,2,3-triazol-4-yl)methyl-4-ethylpiperazine-1-carbodithioate **(32)**

Yellow soild, yield: 88 %, m.p: 70~71 ^o^C. ^1^H NMR (400 MHz, CDCl_3_) δ 8.18 (s, 1H), 7.81 (d, *J* = 7.7 Hz, 2H), 7.57 (d, *J* = 8.3 Hz, 2H), 7.39 (ddd, *J* = 17.9, 8.0, 2.5 Hz, 5H), 6.60 (s, 2H), 5.00 (d, *J* = 2.4 Hz, 1H), 4.80 (d, *J* = 1.5 Hz, 2H), 4.36 (d, *J* = 7.0 Hz, 2H), 4.30 (d, *J* = 2.3 Hz, 1H), 3.93 (d, *J* = 9.9 Hz, 2H), 3.79 (d, *J* = 1.4 Hz, 3H), 3.74 (d, *J* = 1.4 Hz, 6H), 2.54 (s, 4H), 2.46 (q, *J* = 7.2 Hz, 2H), 1.17 – 1.05 (m, 3H). ^13^C NMR (100 MHz, CDCl_3_) δ 195.44, 165.07, 153.68, 145.30, 138.19, 137.24, 134.83, 134.22, 133.34, 129.20, 128.21, 127.38 (d, *J* = 13.4 Hz), 121.35, 121.01, 94.86, 65.18, 63.32, 60.97, 56.12, 52.08, 51.85, 31.47, 29.69, 11.92. HRMS (ESI): calcd C_34_H_39_N_6_O_4_S_2_, [M + Na]^+^ m/z, 659.2466; found, 659.2474.


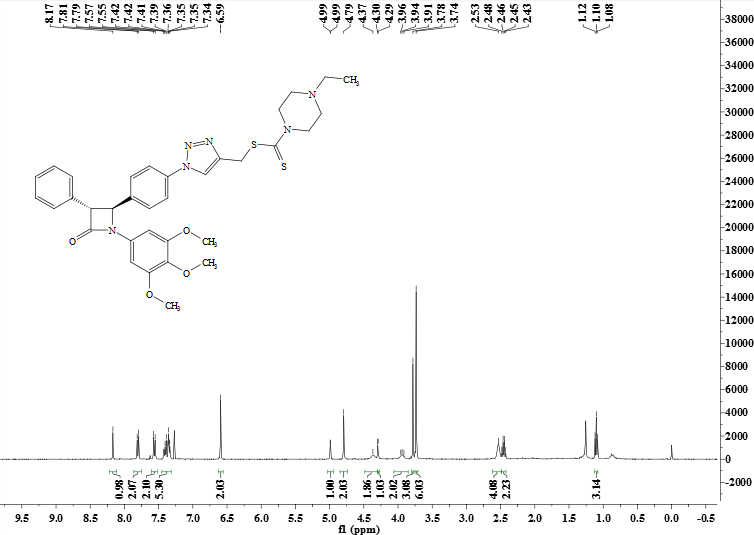


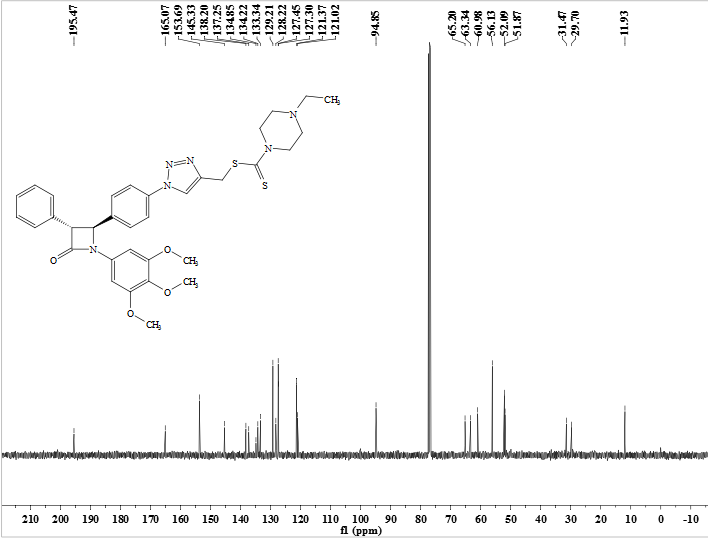


4-(4-azidophenyl)-3-phenyl-3-((trifluoromethyl)sulfonyl)-1-(3,4,5-trime

thoxyphenyl)azetidin-2-one **(35)**

White soild, yield: 47 %, m.p: 187~189 ^o^C. ^1^H NMR (400 MHz, CDCl_3_) δ 7.93 – 7.75 (m, 2H), 7.58 (d, *J* = 8.4 Hz, 2H), 7.51 – 7.40 (m, 3H), 7.07 (d, *J* = 8.5 Hz, 2H), 6.46 (s, 2H), 5.45 (s, 1H), 3.71 (s, 3H), 3.63 (s, 6H). ^13^C NMR (100 MHz, CDCl_3_) δ 154.64, 152.69, 141.10, 134.78, 130.90, 129.90, 129.57, 128.47, 127.89, 126.90, 124.30, 118.35, 94.73, 83.45, 67.37, 59.96, 55.13. HRMS (ESI): calcd C_25_H_22_F_3_N_4_O_6_S, [M + H]^+^ m/z, 563.1215; found, 563.1212.


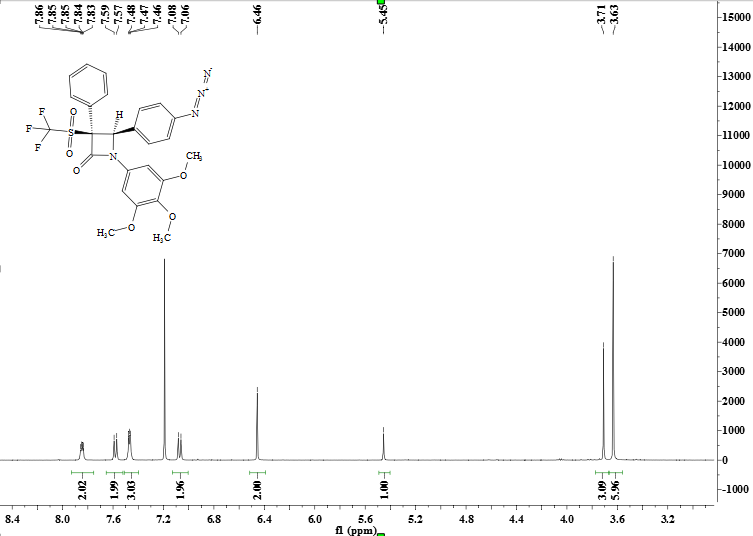


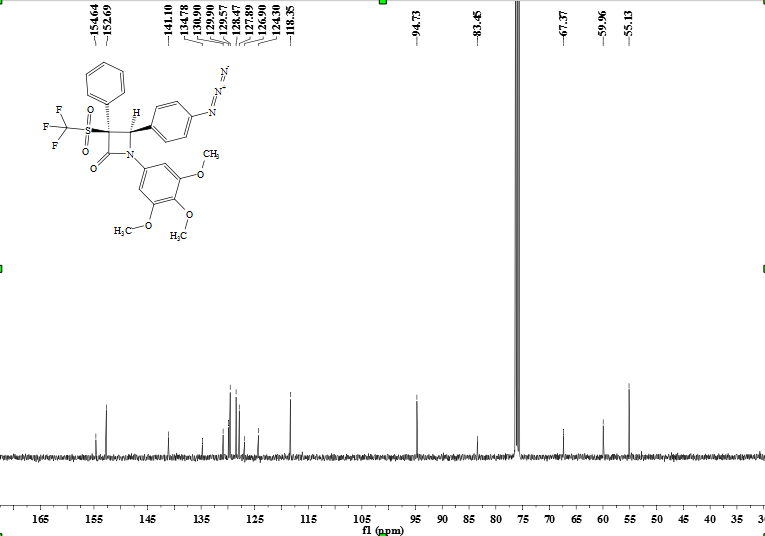


4-(3-azidophenyl)-3-phenyl-3-((trifluoromethyl)sulfonyl)-1-(3,4,5-trimet

hoxyphenyl)azetidin-2-one **(36)**

White soild, yield: 48 %, m.p: 205~207 ^o^C. ^1^H NMR (400 MHz, CDCl_3_) δ 7.84 (dd, *J* = 6.5, 2.8 Hz, 2H), 7.52 – 7.44 (m, 3H), 7.44 – 7.34 (m, 2H), 7.21 (s, 1H), 7.10 (d, *J* = 7.7 Hz, 1H), 6.45 (s, 2H), 5.42 (s, 1H), 3.71 (s, 3H), 3.63 (s, 6H). ^13^C NMR (100 MHz, CDCl_3_) δ 154.72, 152.81, 139.92, 134.96, 130.93, 130.26, 130.05, 129.29, 128.60, 127.96, 126.93, 124.57, 119.72, 118.82, 94.84, 83.63, 67.45, 60.05, 55.24. HRMS (ESI): calcd C_25_H_22_F_3_N_4_O_6_S, [M + H]^+^ m/z, 563.1217; found, 563.1212.


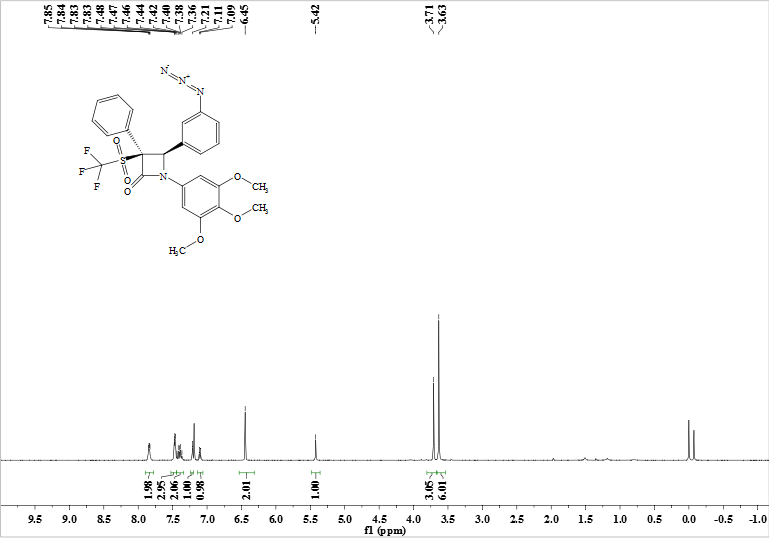


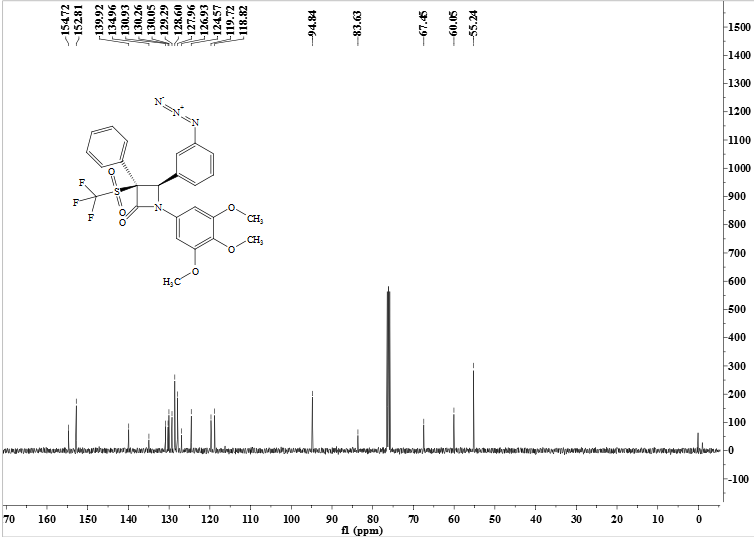


**7. References**

1. Zhang, Z. *et. al*. Chang, X. 3-Thiomorpholin-8-oxo-8H-acenaphtho[1,2-b]pyrrole-9-carbonitrile (S1) Based Molecules as Potent, Dual Inhibitors of B-Cell Lymphoma 2 (Bcl-2) and Myeloid Cell Leukemia Sequence 1 (Mcl-1): Structure-Based Design and Structure−Activity Relationship Studies. *J. Med. Chem.* **54**, 1101-1105 (2011).

2. Da, C. *et. al*. How to Deal with Low-Resolution Target Structures: Using SAR, Ensemble Docking, Hydropathic Analysis, and 3D-QSAR to Definitively Map the αβ-Tubulin Colchicine Site. *J. Med. Chem.* **56**, 7382-7395 (2013).

3. Huang, Z. Y. *et. al*. Stereoselective Synthesis of β‑Lactam-triflones under Catalyst-Free Conditions. *Org. Lett*. **17**, 5610−5613 (2015).
